# Engineering cytokines for cancer immunotherapy: a systematic review

**DOI:** 10.3389/fimmu.2023.1218082

**Published:** 2023-07-06

**Authors:** Yong Fu, Renhong Tang, Xiaofeng Zhao

**Affiliations:** ^1^ State Key Laboratory of Neurology and Oncology Drug Development, Jiangsu Simcere Pharmaceutical Co., Ltd., Nanjing, China; ^2^ Jiangsu Simcere Pharmaceutical Co, Ltd., Nanjing, China; ^3^ Simcere Zaiming Pharmaceutical Co, Ltd., Nanjing, China

**Keywords:** engineering cytokine, fusion protein, polyethylene glycol, immunocytokine, prodrug cytokine, cancer immunotherapy

## Abstract

Cytokines are pivotal mediators of cell communication in the tumor microenvironment. Multiple cytokines are involved in the host antitumor response, but the production and function of these cytokines are usually dysregulated during malignant tumor progression. Considering their clinical potential and the early successful use of cytokines in cancer immunotherapy, such as interferon alpha-2b (IFNα-2b; IntronA^®^) and IL-2 (Proleukin^®^), cytokine-based therapeutics have been extensively evaluated in many follow-up clinical trials. Following these initial breakthroughs, however, clinical translation of these natural messenger molecules has been greatly limited owing to their high-degree pleiotropic features and complex biological properties in many cell types. These characteristics, coupled with poor pharmacokinetics (a short half-life), have hampered the delivery of cytokines via systemic administration, particularly because of severe dose-limiting toxicities. New engineering approaches have been developed to widen the therapeutic window, prolong pharmacokinetic effects, enhance tumor targeting and reduce adverse effects, thereby improving therapeutic efficacy. In this review, we focus on the recent progress and competitive landscape in cytokine engineering strategies and preclinical/clinical therapeutics for cancer. In addition, aiming to promote engineered cytokine-based cancer immunotherapy, we present a profound discussion about the feasibility of recently developed methods in clinical medicine translation.

## Introduction

Cytokines constitute a class of immunoregulatory proteins (generally with a molecular weight lower than 30 kDa) that exert biological functions via the extracellular domains of cell membrane receptors and play imperative roles in maintaining physiological homeostasis and modulating pathophysiological processes (1, 2). The main classes of cytokines engaged in cellular communication include interleukins (ILs), interferons (IFNs), certain members of the tumor necrosis factor (TNF) superfamily, and other effector molecules (3). Dysregulated cytokine production in the tumor microenvironment (TME) is involved in all stages of carcinogenesis as well as in responses to therapy, suggesting that cytokines are crucial in cancer initiation, progression and elimination (4). Thus, cytokine-based therapeutics may be promising candidates for cancer immunotherapy, adding to the list of therapies that include radiotherapy (RT)/chemotherapy and immune checkpoint blockade therapy for cancer (5).

In 1986, interferon α-2b (IFNα-2b) was approved by the U.S. Food and Drug Administration (FDA) for the treatment of hairy cell leukemia (6). As the first biotherapeutic agent authorized, IFN-α provides a mechanistic blueprint for the development of many other cytokines into therapeutics. Subsequently, high-dose IL-2 therapy was authorized by the FDA in 1992 and 1998 for the treatment of metastatic renal cell carcinoma (RCC) and metastatic melanoma, respectively (7). Recently, various cytokines have been investigated in human clinical trials (8). However, the utilization of cytokines more broadly as effective therapeutic agents has been restricted due to their poor drug-like capabilities, extensive systemic effects and pleiotropy, which result in high toxicity and limited effectiveness (9). Indeed, a representative cytokine candidate from Nektar Therapeutics and Bristol Myers Squibb (BMS), polyethylene glycol (PEG)ylated IL-2 (Bempegaldesleukin), recently failed in the first Phase III study, delaying the hoped-for therapeutic resurrection of a pleiotropic cytokine (10, 11).

Why do cytokine-based monotherapies largely fail? The following reasons may explain the problems with native cytokines: short half-life in the circulatory system, low biodistribution, pathway redundancy of the target, a narrow therapeutic window, prohibitive toxicities (especially vascular leakage syndrome, central nervous system toxicity and cardiotoxicity), immunosuppressive effects of cytokines, and context-dependent and pleiotropic qualities (3). With a deeper understanding of target immunobiology and through rapid development of engineering biotechnology, preclinical and clinical investigations of cytokine therapeutics for cancer have markedly expanded, which has greatly facilitated the clinical translation of cytokine therapy. Novel approaches based on cytokine engineering are rapidly developing to localize cytokine activity to cells or tissues of interest and to initiate cytokine activity on demand (12). Notably, on July 28, 2022, ImmunityBio reported that the FDA has accepted a biologics license application (BLA) for the IL-15 agonist N-803 (Anktiva™) for the treatment of non-muscle-invasive bladder cancer (NMIBC) carcinoma *in situ* patients who had not responded to bacillus Calmette-Guerin (BCG) (13–15). The promising clinical data and progress of IL-15 agonists will undoubtedly reinvigorate the field of cytokine engineering for use in cancer therapy. In this review, we mainly concentrate on how engineering strategies and clinical advancements are being exploited for producing cytokines for clinical applications, including the common cytokine receptor γ chain (γc; also designated CD132 or IL-2Rγ) cytokine family (IL-2, IL-15, IL-7 and IL-21), IL-10, IL-12, IL-18, interferons (IFNs) and tumor necrosis factor-α (TNF-α).

## Cytokine engineering strategies for improving therapeutic efficacy

**Figure 1:**
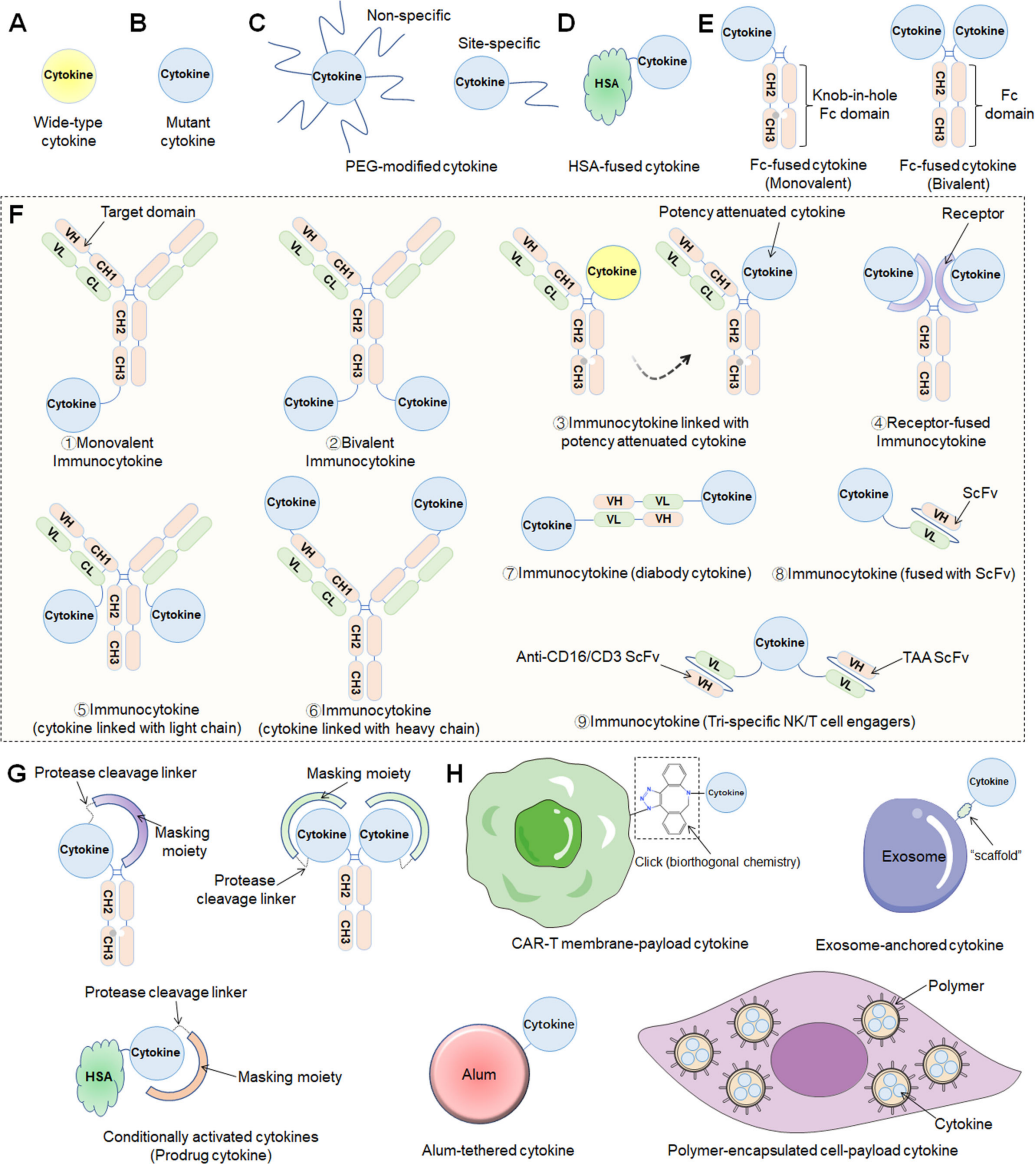
Different engineering approaches to increase the drug-like properties of cytokine-based therapeutics. **(A)** Wild-type cytokine. **(B)** Mutant cytokine. **(C)** PEG modification methods including nonspecific or site-specific chemical conjugation. **(D)** Fusion to the N- and/or C-terminus of HSA. **(E)** Fc-fusion strategies. Monovalent (“knob-in-hole” technology) or bivalent cytokine fused to the N-terminus of the IgG Fc-domain. **(F)** Immunocytokines consist of an antibody fused to a cytokine in various formats (1. Monovalent immunocytokine; 2. Bivalent immunocytokine; 3. immunocytokine with potency attenuated cytokine; 4. Receptor-fused immunocytokine; 5. cytokine linked with light chain; 6. cytokine linked with heavy chain; 7. diabody cytokine-based immunocytokine; 8. immunocytokine (fused with ScFv); 9. Tri-specific NK/T-cell engagers). **(G)** Prodrug cytokines using conditional activation approaches. **(H)** Other approaches, including CAR-T-cell membrane-payload cytokines, exosome-anchored cytokines, alum-tethered cytokines, polymer-encapsulated cell-payload cytokines, *etc.*.

As a crucial subset of cell signaling molecules, cytokines with pleiotropy and complex biology can produce serious adverse effects when administered systemically by inducing overactivation of the immune system (16). Recently, locus mutation has been the foundational engineering strategy to improve cytokine affinity or enhance binding selectivity, which in turn activates cells of interest and related signaling pathways (**Figures 1A, B**).

Cytokine-mediated therapy is subject to poor pharmacokinetic (PK) properties because the small size of the proteins promotes fast vascular extravasation and renal excretion, ultimately leading to a short half-life (8). Thus, through traditional intravenous administration, high-dose and frequent dosing regimens usually contribute to serious toxic side effects due to the narrow therapeutic window. As a result, multiple innovative strategies have been established to overcome delivery difficulties, such as the coupling of cytokines to high molecular weight carrier proteins such as human serum albumin (HSA), fusion with a fragment crystallizable region (Fc) of an immunoglobulin (IgG), or conjugation with polymer——PEG (**Figures 1C, E** **) (** 17, 18). Recently, the aforementioned engineering techniques were extensively utilized in preclinical drug development, and multiple drug candidates have thus been developed. Many researchers named these drugs “supercytokines”, which include “superkine” and “superagonist”.

Cytokine targeting is an urgent problem to be solved because without proper targeting, sufficient therapeutic concentrations cannot be realized in tumors. The category of cytokines developed for targeting are antibody cytokine conjugates termed “immunocytokines” (or “fusokines”), which combine the targeting characteristic of antibodies with the ability of cytokines to trigger a local immune response while also tuning the PKs and pharmacodynamics (PDs) of the biologically active molecule (**Figure 1F**). Notably, a diabody is a novel antibody developed using recombinant DNA technology and consists of two variable heavy light (VH) and two variable light (VL) domains. Each VL domain in a single-chain variable fragment (scFv) is connected with a VH domain via a short linker, resulting in a diabody with two antigen-binding sites pointing in opposite directions (19). In addition, with the development of advanced protein engineering platform technologies, bispecific T/NK cell engagers fused with functional cytokines such as IL-2 and IL-15 have emerged, called trispecific T/NK cell engagers. Recently, diabody-fused cytokines (referred to as “diabody cytokines”) and trispecific T/NK cell engagers have been evaluated in clinical studies, and herein, we classify them as immunocytokines.

Interestingly, other recent approaches to conditionally activate cytokines depending on the TME, referred to herein as “prodrug cytokines”, might reduce systemic adverse events while sustaining antitumor capabilities and allow for enhanced safety and PKs/PDs (**Figure 1G**).

In general, the most recent cytokine engineering strategies can be broadly classified into the following categories: locus mutation, HSA-cytokine fusion, constant antibody fragment (Fc)-cytokine fusion, polymer conjugation, and immunocytokine and prodrug cytokine production (**Figure 1**). In fact, most cytokines used in the clinic are the products of multiple engineering strategies that further improve their efficacy and reduce the number and severity of adverse effects.

### Locus mutation

One of the first engineering strategies used to translate proteins into drugs was the mutation or modification of protein sequences to improve their biological manufacturability (12). More recently, versions of mutated cytokines have been designed to lessen the pleiotropic effects of cytokines. As a consequence, these mutations are employed to either increase or decrease binding affinity to cell receptors, leading to a more selective effect of the engineered cytokine.

### HSA fusion

The short half-life of cytokines significantly limits their clinical application. The neonatal Fc receptor for IgG (FcRn), which recycles IgG and prolongs its half-life in the circulatory system, contributes to effective humoral immunity (20). HSA is recycled through FcRn, which further extends the circulatory half-life of fusion cytokines by reducing the renal clearance rate and salvaging via FcRn (21). Albumin fusion proteins can be relatively easy to synthesize on a large scale, providing more cost-effective methods for enhancing cytokine PKs.

### Fc fusion

An Fc-based fusion protein consists of an immunoglobulin Fc domain that is covalently linked to another protein and is designed to alter the PKs of the active molecule (22). Mechanistically similar to HSA-fused proteins, the Fc domain significantly enhances the plasma half-life of the fusion protein, which improves therapeutic effects due to its interaction with the salvaging FcRn, as well as slower kidney excretion of larger molecules (20). Additionally, the Fc domain engages with Fcγ receptors (FcγR) and complement, which may also result in Fc-mediated various biological effects, including antibody-dependent cellular cytotoxicity (ADCC), antibody-dependent cellular phagocytosis (ADCP), and complement-dependent cytotoxicity (CDC) (23).

### PEG/Polymer modification

PEG is an amphiphilic nonionic synthetic polymer that readily conjugates cytokines to alter their PKs/PDs (24). Interestingly, in contrast to other strategies used for half-life extension, PEG fusion (PEGylation) is a materials-based approach. Currently, PEGylation can be used to achieve site-specific modification, and it can specifically bind an N-terminal α-amino group of proteins or peptides, which makes PEGylated fusion proteins profitable to manufacture at an industrial level (25).

### Immunocytokines

A crucial safety aspect of cytokine therapy is systemic immune activation mediated by diverse receptor expression profiles, resulting in not only on-target but also off-tumor activity. Cytokines can be fused with targeting or functional proteins to modulate their localization or add new functionalities. Accordingly, structurally different formats of immunocytokines have emerged through the fusion of cytokines with antibodies, thereby locating effector molecules in the TEM and expanding the therapeutic window (26).

### Prodrug cytokines

Strategies to conditionally activate the cytokine signaling pathway in the TME might further reduce systemic effects while preserving antitumor function and thus increase safety and PKs/PDs. Recently, leveraging tumor-related proteases or the acidic microenvironment for selective activation of cytokines has emerged as a promising strategy to increase safety and efficiency by limiting peripheral activity and localizing cytokine signaling to the TME. Protease-activatable “masked” cytokines have been illustrated and prepared with various masking domains, including anti-cytokine antibodies, antibody fragments, peptides, and native cytokine receptors (27). Selective activation in the TME depends on the linker design and expression of tumor-specific or abundant proteases, such as matrix metalloproteinase and cathepsins (28). Generally, prodrug cytokines consist mainly of the following four parts (29): (i) a cytokine that mediates antitumor response in the TME; (ii) a masking moiety, also referred to as a blocking moiety, which can be any group that physically prevents cytokines from binding and/or activating receptors *in* or surrounding nontumor tissues *vivo* until it dissociates and is activated in TME; (iii) “half-life extension elements”, which are usually a part of a chimeric polypeptide that improves PKs by changing the size, shape, charge, or the biodistribution, metabolism and renal clearance of the drug; and (iv) a linker, which is an important component of the prodrug cytokine, by providing flexibility between polypeptides so that the blocking element can suppress the activity of the cytokine polypeptide.

### Other engineering approaches

Other engineering approaches have been extensively investigated, including sustained-release cytokine microspheres, microencapsulated engineered cells and exosome-loaded or aluminum hydroxide (alum)-anchored cytokine therapeutics (**Figure 1H**).

## Cytokines used in preclinical and clinical cancer therapy

This section focuses on current breakthroughs in the biology of the abovementioned cytokines (IL-2, IL-15, IL-7, IL-21, IL-10, IL-12, IL-18, IFNs and TNF-α), as well as engineering approaches and competitive landscapes aimed at resurrecting cytokine immunotherapy and promoting their clinical translation for the treatment of cancer.

## IL-2

**Figure 2:**
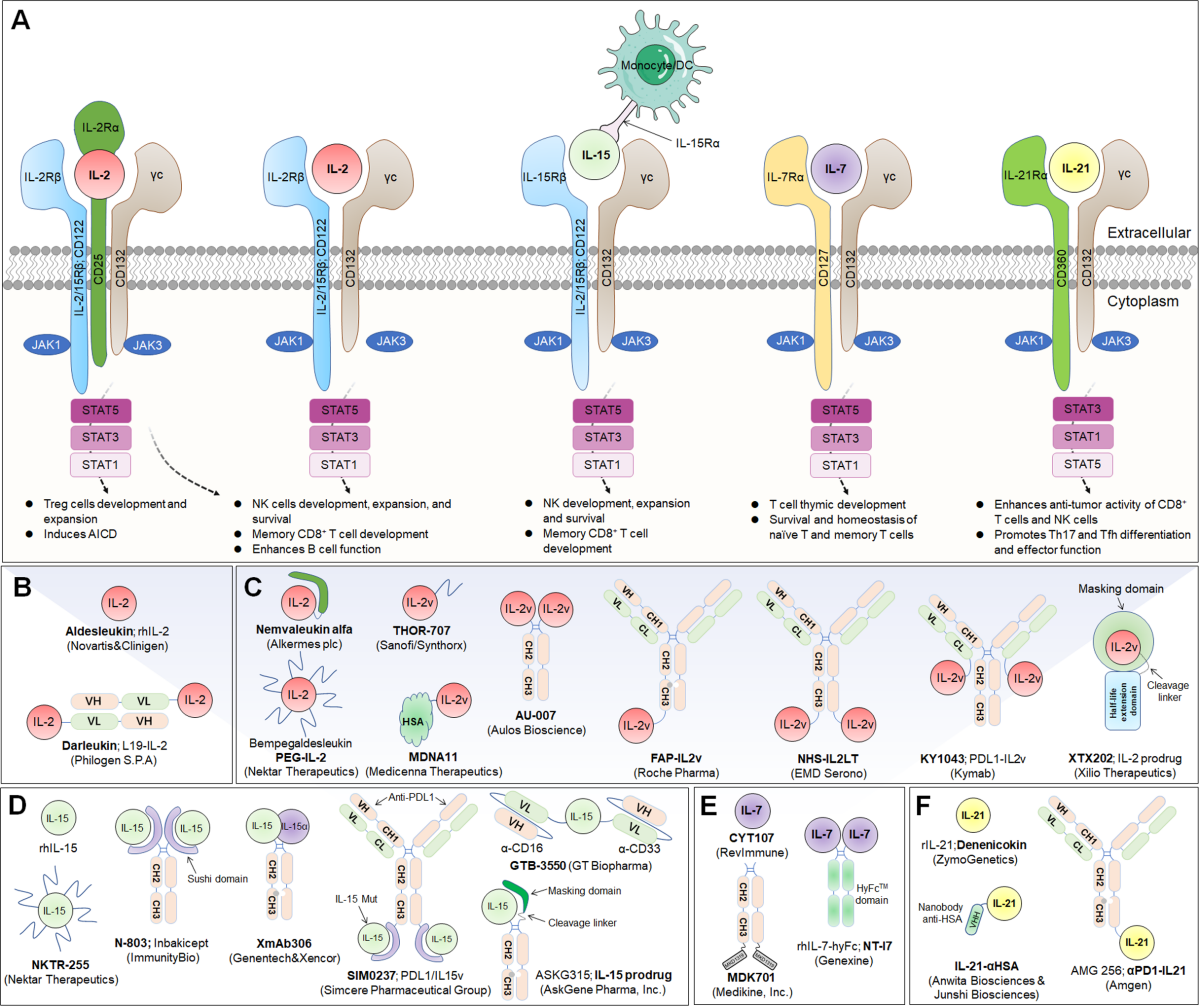
Common γ-chain cytokine (IL-2, IL-15, IL-7 and IL-21) signaling pathways, downstream effector molecules, and representative therapeutic candidates. **(A)** Common γ-chain cytokine (IL-2, IL-15, IL-7 and IL-21) signaling pathways and downstream effects. IL-2 and IL-15 are the only two of these cytokines to have three receptor chains. IL-2 exerts its biological function through high-affinity (IL-2Rαβγ) and intermediate-affinity (IL-2Rβγ) receptors. Engagement of IL-2 with IL-2Rαβγ strongly activates lymphocytes, driving beneficial effects on NK and T cells for development, increased effector function and enhanced B-cell function. The production of autocrine IL-2 can also lead to activation-induced cell death (AICD). Additionally, IL-2Rα is dramatically increased on Treg cells, so IL-2 is uniquely able to promote the expansion and development of immunosuppressive lymphocytes. IL-15 predominantly enhances the effector functions of NK and T lymphocytes without activating Treg cells. IL-7 primarily potentiates T-cell homeostasis, survival and thymic development, including both native and memory phenotypes. IL-21 dominantly promotes Th17 and Tfh differentiation and effector function and enhances the antitumor activity of CD8^+^ T and NK cells. **(B, C)** Representative therapeutic candidates of engineered IL-2 and IL-2 variants. **(D-F)** Representative therapeutic candidates of engineered IL-15, IL-7 and IL-21.

IL-2, which is typically secreted by activated CD4^+^ T lymphocytes, is a prototypically pleiotropic cytokine that promotes T-cell proliferation, survival and differentiation; regulates natural killer (NK) cell natural lethality; and participates in antibody production on B cells (30). The IL-2 receptor (IL-2R) is a heterotrimeric complex with three subunits: IL-2Rα (CD25), IL-2Rβ (CD122), and IL-2Rγ (CD132) chains (31). Among these chains, IL-2Rβ and IL-2Rγ (IL-2Rβγ) constitute an intermediate-affinity dimeric receptor (Kd~10^-9^ M), while IL-2Rα, IL-2Rβ and IL-2Rγ (IL-2Rαβγ) constitute a high-affinity trimeric receptor (Kd~10^-11^ M) (32). Dimeric intermediate-affinity IL-2R (IL-2Rβγ) is expressed primarily on CD4^+^ and CD8^+^ memory T lymphocytes and CD56^dim^ NK cells; trimeric high-affinity IL-2R (IL-2Rαβγ) is expressed mainly on regulatory T (Treg) cells, activated CD4^+^ and CD8^+^ effector T lymphocytes, some NK cells, NK T cells, and group 2 innate lymphoid cells (ILC2s) (32). Once IL-2 specifically binds to IL-2R, its configuration changes, initiating the JAK1-JAK3-STAT5, PI3K-AKT-mTOR, and MAPK signaling pathways, inhibiting apoptosis, inducing cell proliferation and differentiation, and producing a variety of biological effects (33). Proleukin**^®^** (aldesleukin) was the first recombinant IL-2 immunotherapeutic agent to be developed, jointly by Novartis and Clinigen, but its clinical application is greatly limited due to its short half-life, narrow therapeutic window, profoundly toxic side effects, and stimulation of Treg cell activation (34) (**Figure 2A**).

To achieve the beneficial activation of cytotoxic T lymphocytes to enhance cancer immunotherapy, a variety of engineering strategies have concentrated on altering the binding affinity of IL-2 to distinct receptor subunits (**Table 1**; **Figures 2B, C**). Subsequently, multiple new-generation IL-2 variants have been developed. In 2000, Bayer designed **Bay50-4798**, which selectively binds IL-2Raβγ, thereby reducing IL-2Rβγ^+^ NK cell-mediated toxicity, but the ultimate clinical benefit was not better than that of traditional IL-2 (35). In two ground-breaking studies published in 2005 and 2012, the prestigious scientist K. Christopher Garcia and colleagues analyzed the structure of the IL-2 quaternary complex with its different IL-2 receptors (36) and elucidated the underlying molecular mechanism by which IL-2 sensitizes T cells (37), which facilitated the use of biased IL-2 variants in clinical trials.

**Table 1:** Characteristics of engineered IL-2 variants.

Name	Engineered approaches	Mechanism of actin	Tumour type	Stages	Institutions/References
Proleukin (aldesleukin)	Recombinant human IL-2 from engineered *E. coli* strain	High-dose IL-2	Metastatic RCC and melanoma	Launched	Novartis; Clinigen
Bay50-4798	Locus mutation	IL-2-specific agonist	Advanced melanoma and renal cancer	Phase I	Bayer AG
Bempegaldesleukin (NKTR-214)	PEG modification (six cleavable PEG groups); similar to prodrug	Long-acting	Solid tumours Melanoma, RCC, bladder cancer	Phase III	Nektar Therapeutics; Bristol Myers Squibb
THOR-707 (SAR444245)	Locus mutation with PEG modification (one noncleavable PEG group)	Selective activation of CD8^+^ T cells	Advanced or metastatic solid tumours	Phase I/II	Sanofi/Synthorx
MDNA11	Locus mutation with HSA-fusion IL-2 “superkine”	Biased agonist for CD122 (IL-2Rβ) and long-acting	Solid tumours	Phase I	Medicenna Therapeutics
SHR-1916	Locus mutation and PEG modification	Promoted the amplification of NK and T lymphocytes	Advanced malignant tumours	Phase I	Hengrui Pharmaceuticals
Nemvaleukin alfa (ALKS 4230)	Fusion IL-2Rα domain; Circularly permuted IL-2v–IL2Rα fusion protein	Selectivity for NK cells	Solid tumours	Phase III	Alkermes plc
STK-012	Locus mutation with PEG modification	Partial agonist targeting activated T cells	Solid tumours	Phase Ia/Ib	Synthekine
AU-007	Locus mutation; Selective to the CD25-binding; computationally designed	Biased IL-2; leveraged the body’s own IL-2 to enhance antitumour effects	Unresectable locally advanced or metastatic tumours	Phase I/II	Aulos Bioscience
NL-201 (Neo-2/15)	Agonist of the IL2Rα-independent IL-2/IL-15 receptors; computationally designed	Selectively stimulated expansion and tumour infiltration of cytotoxic NK CD8^+^ T lymphocytes	R/R cancer	Phase I	Neoleukin Therapeutics
HuKs-IL2 (EMD 273066)	Immunocytokine; fused with EpCAM	Targeted EpCAM-positive advanced solid tumours	Solid tumours	Phase I/II	EMD Serono
Hu14.18-IL2 (EMD 273063)	Immunocytokine; fused with GD2 fusion	Targeted GD2-positive advanced solid tumours	Melanoma; Relapsed or Refractory neuroblastoma	Phase II	EMD Serono
DI-Leu16-IL2	Immunocytokine; anti-CD20 antibody fused to natural IL-2	Stimulated the immune system	B-cell NHL	Phase I	Alopexx Oncology, LLC
RO6895882 (CEA-IL2v)	Immunocytokine; Locus mutation and fused with CEA	Targeted CEA-positive advanced solid tumours	Metastatic solid tumours	Phase I	Roche Pharma
RO6874281 (FAP-IL2v)	Immunocytokine; Locus mutation and fused with FAP	Targeted FAP-positive advanced solid tumours	Metastatic RCC and melanoma	Phase Ib	Roche Pharma
L19-IL-2 (Darleukin)	Immunocytokine; Locus mutation and fused with L19	Targeted human vascular	Solid tumours; DLBCL	Phase I/II	Philogen S.P.A
F16-IL-2	Immunocytokine; Locus mutation and fused with F16	Binded to tumour cells expressing tenascin-C	Solid tumours	Phase Ib/II	Philogen S.P.A
NHS-IL2LT (EMD 521873)	Immunocytokine; Locus mutation fused with an antibody (NHS76)	Targeted the necrotic core of tumours	Lung cancer; NHL	Phase I	Merck KGaA
CUE-101	Immunocytokine; Locus mutation and fused with HPV16 E7 peptide	Activated HPV16-specific CD8^+^ T	HNC	Phase I	Cue Biopharma
RG6279 (PD1-IL2v)	Immunocytokine; Locus mutation and fused with PD-1 blocking antibody	Tumour-targeted and selective activation of CD8^+^ T lymphocytes	Solid tumours	Phase Ia/Ib	Roche Pharma
KY1043	Immunocytokine; Locus mutation and fused with PD-L1 blocking antibody	Tumour-targeted and selective activation of T lymphocytes	Solid tumours	IND application in 2021	Kymab
WTX-124	Conditionally activated wild-type IL-2 prodrug	Selective tumour release and stimulate the immune system	Advanced solid tumours	Phase I/Ib	Werewolf Therapeutics
XTX202	Tumour-activated mutant IL-2 prodrug; not binding IL-2Rα	A tumour-activated IL-2; potently stimulate CD8^+^ effector T and NK cells	Solid tumours	Phase I/II	Xilio Therapeutics
ODC-IL2	Tumour-selective IL-2 prodrug; linked to a cytokine-inhibitory moiety	Local released and triggered antitumour immune response	Solid tumours	Preclinical	Trutino Biosciences
IL-2-payload ARPE-19	Cytokine delivery platform	Polymer-encapsulated human epithelial cells to produce natural cytokines	Solid tumours	Preclinical	Nash et al. (76)

**NKTR-214** (bempegaldesleukin) is a representative PEGylated IL-2 with six slowly released functional groups that systematically deliver and preferentially bind to the IL-2Rβγ subunit but not the IL-2Rα subunit, thus promoting the effect of IL-2 on effector NK and T lymphocytes but not Treg cells (38). This mechanism of action resulted in selective expansion of effector T lymphocytes and inhibition of Treg cell proliferation in preclinical animal models (39). By modifying aldesleukin with PEG, the resulting compound NKTR-214 showed an effective reduction in severe toxic and other side effects and prolonged cancer onset compared to unmodified aldesleukin. However, on March 14, 2022, based on data from the Phase III PIVOT IO-001 study (NCT03635983), BMS and Nektar Therapeutics disclosed that anti-PD-1 antibody (Opdivo^®^) in combination with Bempegaldesleukin in the treatment of metastatic melanoma did not show key differences in the primary endpoints of progression-free survival (PFS) and overall response rate (ORR) compared with those of Opdivo^®^ monotherapy (40). Additionally, according to the findings from PIVOT IO-001, the companies decided to terminate enrolment and unblind the ongoing Phase III PIVOT-12 study for patients with adjuvant melanoma, which was designed to evaluate differences in the dual therapy of Bempegaldesleukin combined with Opdivo^®^ versus Opdivo^®^ monotherapy in participants with high risk for recurrence after resection of melanoma (NCT04410445) (41).

**THOR-707** (SAR444245) is a novel recombinant human IL-2 (rhIL-2) molecule that is irreversibly bound to a PEG moiety via click chemistry to block α-chain binding while maintaining appropriate affinity for IL-2Rβ/γ subunits (42). In preclinical studies, THOR-707, administered either as a single agent or in combination with an anti-PD-1 antibody, showed clear antitumor benefits without inducing severe side effects (43). A HAMMER Phase I/II trial documented that THOR-707 had good tolerability and showed antitumor effects in cancer patients, including those who were previously treated with checkpoint inhibitors. Combination therapy with pembrolizumab and cetuximab leveraged the effects of SAR44245 on CD8^+^ T and NK cells (NCT04009681) (44).

**MDNA11**, designed by Medicenna Therapeutics, is an IL-2-based “Superkine” fused with human recombinant albumin designed to increase the cytokine half-life and minimize dosing requirements without sacrificing efficacy or safety (45). Notably, this next-generation IL-2 drug preferentially binds CD122 (and shows no CD25 affinity) on immune cells and acts as a powerful switch for activating and proliferating tumor-killing effector NK and T lymphocytes to fight cancer (46). A Phase I/II ABILITY trial is ongoing to evaluate the safety, PKs/PDs, and antitumor properties of various dosages of MDNA11 in participants with advanced solid tumors (NCT05086692).

**SHR-1916** is a new PEG-modified and site-mutated IL-2 molecule independently developed by Hengrui Medicine. It can activate the JAK1-JAK3-STAT5 signaling pathway, boost the proliferation of NK and CD8^+^ T lymphocytes, and exert antitumor effects (47). A Phase I clinical study of SHR-1916 is under underway based on clinical approval to assess its tolerability, safety, PK characteristics and preliminary efficacy in individuals with advanced malignant tumors (NCT04842630) (47).

Another clinically investigated IL-2-related candidate agent is **ALKS 4230** (Nemvaleukin alfa, from Alkermes plc), which is a fusion protein composed of a circularly permuted IL-2 with an extracellular IL-2Rα domain that was designed for selective activation of effector lymphocytes with intermediate affinity for IL-2R (48, 49). Currently, a Phase III study (ARTISTRY-7) of ALKS 4230 combined with pembrolizumab for participants with platinum-unresponsive epithelial ovarian cancer is ongoing (NCT05092360) (50).

**AU-007**, designed by Aulos Bioscience, is a computationally developed, human IgG1 monoclonal antibody with LALA mutations in the Fc domain and is highly selective for the CD25-binding portion of IL-2 (51). Interestingly, AU-007 shows a different mechanism of action than other IL-2 therapies being developed, as it leverages the body’s own IL-2 to increase its innate antitumor immune effects. This increased effect was achieved by preventing IL-2 secreted from effector T cells from binding to trimeric receptors on Treg cells while still allowing IL-2 to bind to and induce the expansion of the effector T cells (52). A Phase I/II dose-escalation and expansion trial of AU-007 in participants with unresectable locally advanced or metastatic cancer is ongoing (NCT05267626) (52).

According to the report published in *Nature* magazine in January 2019, **NL-201** (Neoleukin-2/15; Neo-2/15), developed by Neoleukin Therapeutics, is the first computationally designed *de novo* CD25-independent IL-2/IL-15 receptor agonist that specifically boosts the proliferation and tumor infiltration of NK and CD8^+^ T lymphocytes, thus improving antitumor performance (53). A first-in-human (FIH) Phase I trial of NL-201 as a monotherapy and combination therapy with pembrolizumab for participants with advanced cancer is ongoing (NCT04659629) (54).

### IL-2-based immunocytokines

To further enhance the transmission of IL-2 to tumor lesions and improve its therapeutic effect, several groups have combined IL-2/IL-2R mutations with antibody-mediated tumor-targeted delivery strategies (**Table 1**; **Figures 2B, C**). IL-2 fused with tumor-associated antigen (TAA)-targeted antibodies has been exploited, and these drugs can be classified into two main categories: cell-surface antigens, including epithelial cell adhesion molecule (EpCAM), ganglioside D2 (GD2), CD20, and carcinoembryonic antigen (CEA), and tumor extracellular matrix (ECM) targets, such as angiogenesis-related extra domain A/B (EDA/EDB) of fibronectin, tenascin-C, and collagen.

Humanized KS-IL-2 (**HuKs-IL2**; EMD 273066) is an immunocytokine with specific affinity for EpCAM fused at the Fc end to two IL-2 molecules (55), and it has been investigated in combination with cyclophosphamide in a Phase I/II trial of participants with recurrent EpCAM^+^ ovarian cancer, prostate cancer, colorectal cancer (CRC) or non-small cell lung cancers (NSCLC) (NCT00132522) (56). Using the same molecular construction strategy, EMD Serono developed Hu14.18-IL2 (EMD 273063), which is composed of anti-GD2 antibody 14.18 and IL-2 molecule, and Phase I/II studies were performed to determine its effect on patients with recurrent melanoma (NCT00590824) (57) or with relapsed/refractory (R/R) neuroblastoma (NCT01334515) (58). Based on compelling preclinical data, the maximal tolerated dose (MTD) of Hu14.18-IL2 was established to be 12 mg/m^2^/day (59).

In a Phase I dose escalation study, subcutaneous administration of **DI-Leu16-IL2** (anti-CD20 antibody fused to wild-type IL-2) resulted in complete response (CR) in multiple cancer patients with minimal or no adverse events (60). **CEA-IL2v** (RG7813; RO6895882; cergutuzumab amunaleukin) and **FAP-IL2v** (RG7461; RO6874281; simlukafusp alfa) are CEA/fibroblast activating protein (FAP)-targeted immunocytokines designed by Roche Pharma with a novel monomeric IL-2 variant (IL2v) that completely lacks CD25 binding capacity (61). The IL2v component is engineered by structurally changing critical residues in the IL-2Rα interface (F42A, Y45A, and L72G) to prevent binding to IL-2Rα while preserving affinity for IL-2Rβγ (62, 63). Moreover, in multiple clinical Phase I trials, CEA-IL2v has been assessed for use against advanced and/or metastatic solid tumors (NCT02004106) (64). In parallel, FAP-IL2v has been investigated for use against metastatic melanoma (NCT03875079) and metastatic RCC (NCT03063762) and with trastuzumab or cetuximab (NCT02627274) or atezolizumab (NCT03386721) for use against metastatic pancreatic ductal adenocarcinoma (NCT03193190).

**Darleukin** (L19-IL2), developed in collaboration with academia and Philogen S.p.A., is a human vasculature-targeting monoclonal antibody (L19) fused to IL-2, and L19 targets the EDB region, which is contained in the ECM-associated fibronectin (B-FN) isoform (65, 66). A Phase I/II trial of L19-IL2 combined with rituximab against R/R diffuse large B-cell lymphoma (DLBCL) is ongoing (NCT02957019). Meanwhile, Philogen S.p.A. developed an F16-IL-2 immunocytokine in which the human monoclonal antibody fragment F16 scFv against EDA of tenascin-C was fused by linking to rhIL-2, and it showed potential immunostimulatory and antineoplastic activities (67).

Another clinical exploration of IL-2-based immunocytokines is **NHS-IL2LT** (EMD 521873; Selectikine; LT indicates low toxicity), developed by Merck KGaA, which contains an IL-2v (D20T) fused with an antibody (NHS76) that targets the necrotic core of tumors (68). NHS76 shows high affinity for both double- and single-stranded DNA (dsDNA/ssDNA), thus targeting exposed DNA and directing the cytokine to regions of tumor necrosis and apoptosis (69). To date, NHS-IL2LT has shown the lowest toxicity among all IL-2 variants in clinical trials (70).

To solve the problem of drug resistance to immune checkpoint monoclonal antibodies and further strengthen synergistic effects and targeting precision, immune cytokines based on immune checkpoint monoclonal antibodies have been developed. PD1-IL2v (**RG6279**; RO7284755, from Roche) is an antibody protein fusion that blocks PD-1 and is combined with an engineered IL-2v that induces immune-modulating activity (71). **KY1043** (from Kymab; acquired by Sanofi in 2021) is a differentiated immunocytokine consisting of a PD-L1 antibody fused to an IL-2v (retaining the IL-2Rα affinity) designed to enhance antitumor immune responses by blocking the interaction between PD-L1 and PD-1 and activating antigen-experience T lymphocytes while reducing IL-2 activity to improve tolerability (72).

### IL-2-based prodrugs

Tumor-selective IL-2 prodrugs have also recently attracted considerable interest from different research groups for the purpose of increasing intratumoral IL-2 abundance and reducing toxic side effects caused by systemic administration via injection (**Table 1**; **Figure 2C**). Its activity is based on a protease-cleaved substrate, which is sensitive and specific to proteases and preferentially overexpressed in tumors. **WTX-124** is a novel systemically administered, tumor-selective IL-2 prodrug candidate consisting of a wild-type IL-2 cytokine tethered to an inactivation domain to prevent activation in nontarget tissue, a tumor protease-specific linker to allow activation in the TME and a half-life extension element to improve tumor exposure (73). At present, WTX-124 exhibits favorable antitumor abilities with beneficial PKs and tolerability profiles in preclinical experiments. In July 2022, Werewolf Therapeutics started enrolling patients in a FIH Phase I, multicenter study of WTX-124 administered as a single agent and in combination with pembrolizumab to participants with advanced solid tumors (NCT05479812).

**XTX202** is a conditionally activated, βγ-chain biased (non-α-chain), engineered IL-2 molecule that is masked with a protein domain to limit binding activity until it is cleaved by TME-specific proteases. In preclinical studies, XTX202 was activated in a protease-dependent manner, inhibited tumor growth and had favorable tolerability (74). Xilio Therapeutics recently sponsored a Phase I/II clinical study for assessing XTX202 in the treatment of solid tumors (NCT05052268) (75).

Several other novel strategies have been used to engineer IL-2 prodrugs. For example, Nash et al. designed a clinically applicable cytokine delivery platform consisting of polymer-encapsulated human retinal pigmented epithelial cells, called ARPE-19, that produces natural cytokines, such as IL-2, and it significantly eliminated peritoneal tumors in ovarian and colorectal mouse models (76).

## IL-15

Interleukin-15 (IL-15) is a well-documented potent immunoregulator that is typically produced by monocytes and macrophages and can stimulate the proliferation of immune cells such as NK cells, CD8^+^ T lymphocytes, and γδ T cells (77). The biological functions of IL-15 are similar to those of IL-2, and their receptors share the same β chain and γ chain, while they also carry their own distinct α-receptors, IL-15Rα (CD215) and IL-2Rα (CD25), respectively (78). Independent of the IL-15Rβ and γ chains, IL-15Rα is normally expressed by antigen-presenting cells (79). It specifically binds to IL-15, forms an immunological synapse with the IL-15Rβγ chain on peripheral effector NK cells and T lymphocytes, and activates the JAK-STAT signaling pathway in NK cells and T cells (80). Due to its powerful effects on cytolytic NK and T cells without causing the expansion of suppressive Treg cells, IL-15 has recently emerged as an alternative to IL-2 in cancer therapy (81) (**Figure 2A**). Structurally, IL-15Rα comprises a Sushi domain (amino acids 1-65) that interacts with IL-15 and plays an essential role in the biological function of IL-15 (82). However, similar to other cytokines, because of a “cytokine sink”, native IL-15 is characterized by a short half-life and low monomeric bioactivity (83). Moreover, low systemic exposure and poor bioefficacy are associated with dose-dependent systemic toxicity. Therefore, the development of IL-15-based drugs has focused on optimizing their pharmaceutical properties by increasing their biological activity and half-life.

Currently, several IL-15-based superagonists with different structures are in clinical development (**Table 2**; **Figure 2D**). For example, **N-803** (formerly ALT-803; Anktiva^®^), an IL-15 superagonist, consists of an IL-15 mutant (IL-15N72D) bound to the IL-15Rα/IgG1 Fc fusion protein and directly and specifically boosts the proliferation and activation of NK and CD8^+^ T lymphocytes through βγ receptor binding without activating Treg cells (84). Compared to native IL-15, N-803 shows better PK properties, a longer lymphoid tissue retention time, and higher antitumor activity *in vivo*. In a recent QUILT-3.032 Phase II/III trial (NCT03022825) (13), a total of 160 individuals with BCG-unresponsive NMIBC were enrolled, and the overall survival (OS) rate for bladder cancer was 99% at two years. For CIS patients, the complete response (CR) rate was 71%, with a 24.1-month median response duration, and the papillary disease-free survival (DFS) rate was 53% at 18 months. At the 2-year follow-up, more than 90% of patients avoided cystectomy. N-803 in combination with BCG outperformed other current intravesical and systemic treatments for BCG-refractory NMIBC in terms of efficacy and safety. Based on the aforementioned study, the FDA accepted the biologics BLA for N-803 in July 2022 for the treatment of NMIBC carcinoma *in situ* patients who had not responded to BCG. However, in May 2023, the FDA has delayed this approval decision due to deficiencies identified during a pre-licensing inspection of the manufacturer’s third-party contracting company.

**Table 2:** Characteristics of engineered IL-15 variants.

Name	Engineered approaches	Mechanism of actin	Tumour type	Stages		Institutions/References
N-803 (Inbakicept; ALT-803)	IL-15(N72D) bound to IL-15Rα sushi domain linked to IgG1-Fc domain; Locus mutation	Reconstituted and activated human NK cells	Solid tumours	BLA submitted		ImmunityBio
NKTR-255	PEG-conjugated; IL-15Rα-dependent rhIL-15 agonist	Increased the expansion and survival of cancer-killing NK and memory CD8^+^ T lymphocytes	Haematologic malignancies; Solid tumours	Phase Ib/II		Nektar Therapeutics
SOT101 (SO-C101) (RLI-15)	Fusion protein of IL-15 linker to IL-15Rα sushi domain	Reconstituted human NK and T lymphocytes	Solid Tumours	Phase Ib/II		SOTIO Biotech AG
SHR-1501	IL-15 cross-linked with the high-affinity binding sushi domain of IL-15Rα with human Fc-fused protein	Activated and increased the levels of NK cells and memory CD8^+^ T lymphocytes	Advanced malignancie; bladder cancer	Phase I/II		Hengrui Medicine
BNZ-1	PEGylated peptide antagonist	Designed to specifically bind the γ-chain receptors and selectively blocked IL-2, IL-15 and IL-9 signalling	Cutaneous T-cell lymphoma	Phase I		Bioniz Therapeutics
NIZ985	Heterodimeric IL-15 (hetIL-15) IL-15/sIL-15Rα complex; secreted fusion protein	Promoted cytotoxic lymphocyte proliferation, killing function	Metastatic solid cancer	Phase I		Novartis Pharmaceuticals
XmAb306	Potency-reduced IL-15/IL-15Rα-Fc fusion protein	Stimulated NK cells and T lymphocytes	R/R multiplemyeloma; Solid tumours	Phase I		Genentech; Xencor
BJ-001	Immunocytokine; integrin/IL-15 fusion protein with IgG1 Fc	Tumour-targeted; activated NK and T cells	Solid tumours	Phase Ia/Ib		BJ Bioscience
SIM0237	Immunocytokine; αPD-L1/IL-15v fusion protein	Tumour-targeted; effectively reduced T/NK proliferation to increase therapy window	Solid tumours	IND application in 2022		Simcere Pharmaceutical Group
PF-07209960	Immunocytokine; αPD1/IL-15v fusion protein	Stimulated the expansion of NK cells, cytotoxic T lymphocytes (CTLs) and memory T cells	Advanced or metastatic solid tumours	Phase I		Pfizer
GTB-3550	Tri-Specific Killer Engager (TriKE^®^); αCD16/IL-15/αCD33 tri-specific scFv recombinant fusion protein	Activated NK cells	R/R AML; high-risk MDS	Phase I/II		GT Biopharma
ASKG315	Engineered Fc-IL-15 proteolytically activated cytokine prodrug	Designed to improve PKs and provided a wider therapeutic window	Advanced solid tumours.	Phase I		AskGene Pharma, Inc.
ASKG915	A tumour-selective and *cis*-potentiated PD-1 antibody fused with IL-15	PD-1 blockage; improved PKs, provided a wider therapeutic window and enhanced therapeutic potentials	Advanced solid tumours.	IND application in 2023		AskGene Pharma, Inc.

**NKTR-255** is an experimental PEG-conjugated IL-15R agonist aimed at increasing the intrinsic anticancer capacity of the immune system by promoting the survival and expansion of tumor-killing NK cells as well as memory CD8^+^ T lymphocytes (85, 86). NKTR-255 engages the complete IL-15 receptor complex (IL‐15Rα/IL‐15Rβγ) to improve the formation of long-term immunological memory, perhaps leading to persistent antitumor effects (87). NKTR-255 was developed to overcome the challenges of natural IL-15, which must be administered in high dosages due to quick clearance, limiting its effectiveness because of toxicity and inconvenience of use. NKTR-255 is now being studied in a Phase I trial for participants with R/R non-Hodgkin’s lymphoma (NHL) and multiple myeloma (MM) (NCT04136756) (88), and a Phase Ib/II trial is ongoing for evaluating NKTR-255 therapeutic effects in combination with ERBITUX^®^ for R/R colorectal cancer (CRC) and squamous cell carcinoma of the head and neck (SCCHN) (NCT04616196) (89).

**SOT101** (also known as RLI-15 and formerly as SO-C101) is an IL-15 superagonist fused to the IL-15Rα chain and designed to selectively bind only to cytotoxic NK and T lymphocytes but not to other cell types to minimize adverse events when administered subcutaneously. In addition, the PKs of SOT101 enable it to ideally stimulate NK and T lymphocytes through pulses in cytokine concentration not tonic stimulation (90). In a Phase I/Ib trial, the AURELIO-03 trial, SOT101 demonstrated a favorable safety profile and encouraging efficacy both when given as a monotherapy and in combination with KEYTRUDA^®^ (pembrolizumab) (NCT04234113) (91). Subsequently, a Phase II AURELIO-04 trial of SOT101 in combination with pembrolizumab (NCT05256381) is ongoing to assess its efficacy and safety for patients with selected advanced/refractory solid tumors (90).

**BNZ-1** is a PEGylated peptide antagonist designed to specifically bind γ-chain receptors and selectively block IL-2, IL-15 and IL-9 signaling (92). Preliminary Phase I data clarified that BNZ-1 was well tolerated and inhibited IL-2 and IL-15 activity, leading to clinical improvement in participants with cutaneous T-cell lymphoma (CTCL) by rejuvenating anti-lymphoma immunity and reducing inflammatory responses (NCT03239392) (93).

**NIZ985** is a recombinant heterodimer of two polypeptide chains—physiologically active IL-15 and IL-15Rα—which together are named heterodimeric IL-15 (hetIL-15) (94). In preclinical studies, NIZ985 promoted cytotoxic NK and T lymphocyte expansion, tumor-killing function, and tumor infiltration, leading to significant anticancer ability. A recent FIH Phase I study was performed to assess the safety, PKs, and immune efficacy of NIZ985 in participants with metastatic or unresectable solid tumors (NCT02452268) (95). Subcutaneous NIZ985 three times a week was well tolerated in patients with advanced solid tumors and triggered a favorable immune response paralleling those identified in preclinical observations, manifesting as the induction of IFN-γ and amplification of cytotoxic lymphocytes.

**XmAb306** (XmAb24306; RO7310729, from Genentech, a member of the Roche Group) is a potency-reduced IL-15/IL-15Rα fusion protein with an XmAb^®^ bispecific Fc domain (96). Currently, two Phase I clinical trials to evaluate XmAb24306 as a monotherapy and in combination with atezolizumab or daratumumab in participants with locally advanced/metastatic solid tumors (NCT05243342) or R/R M/M (NCT04250155) are ongoing. No dose-limiting toxicities (DLTs) or severe drug-related side effects have been identified to date.

### IL-15-based immunocytokines

IL-15-based immunocytokines are also being developed in the clinic to further improve targeting (**Table 2**; **Figure 2D**). Recently, the Simcere Pharmaceutical Group developed a PD-L1/IL-15v bifunctional immunocytokine (**SIM0237**) that has best-in-class potential with improved tumor control and low safety risk. It blocks PD-1/PD-L1 signaling and delivers IL-15 directly to the TME for full activation of CD8^+^ T lymphocytes. The IL-15 pharmacological profile was fine-tuned through protein engineering. According to preclinical data, SIM0237 effectively reduced T/NK proliferation to increase the therapy window *in vitro* and significantly inhibited tumor growth in an NCI-H292-hPBMC xenograft model *in vivo*. In October 2022, the Simcere Pharmaceutical Group submitted an Investigational New Drug Application (IND) application for SIM0237 to the FDA.

**PF-07209960** is a fusion protein composed of PD-1 fused to an IL-15 mutant designed to demonstrate potential immune checkpoint inhibition, immunomodulation, and antitumor activity. PF-07209960 is produced by Pfizer, which is currently enrolling participants for a Phase I clinical trial (NCT04628780) (97).

**GTB 3550** (OXIS 3550) is a single-chain, triple compound-specific scFv recombinant fusion protein (Tri-specific NK cell engagers; TriKE^®^) composed of anti-CD16 and anti-CD33 antibodies and an IL-15 variant developed by GT Biopharma (owner of Oxis Biotech) for the treatment of patients with R/R acute myelogenous leukemia (AML) and high-risk myelodysplastic syndrome (MDS) (98–101). The candidate was also intended to be investigated in CD33^+^ hematopoietic malignancies in addition to MDS and AML. The drug was developed based on the hypothesis that GTB-3550 TriKE^®^ induces NK cell function by targeting malignant cells as well as CD33^+^ myeloid-derived suppressor cells (MDSCs). Because CD16 is the most potent activating receptor on NK cells, GTB-3550 may elicit a targeted anti-CD33^+^ tumor response. Interim results from the current Phase I/II clinical trial showed that patients treated with GTB-3550 presented with up to a 63.7% downregulation in bone marrow blast levels, suggesting a clinical benefit (NCT03214666). Due to the discovery of second-generation camelid single-domain antibody technology, which presents several advantages over traditional IgG monoclonal antibodies, GTB-3550 development was halted, and a related clinical study was terminated. In September 2021, GT Biopharma announced the advancement of the second-generation camelid-derived nanobody TriKE agent product **GTB-3650** into IND-enabling studies.

### IL-15-based prodrugs

IL-15-based prodrug candidates have also attracted the attention of scientists (**Table 2**; **Figure 2D**). **ASKG315** (ASKG215β) is an engineered proteolytically activated Fc-IL-15 cytokine prodrug designed to show better PKs and provide a wide therapeutic window. It was independently developed by AskGene Pharma, an overseas subsidiary of Jiangsu Aosaikang Pharmaceutical Co., Ltd (102). To fully harness the therapeutic potential of cytokine molecules, AskGene created a novel cytokine prodrug platform (SmartKine^®^), wherein cytokine molecules avoid the cytokine sink when systematically injected and are activated at the site of the lesion. The IL-15-based prodrug candidate ASKG315 has shown sustained PKs from 7 to 15 days in animal models. In the third quarter of 2022, two Phase I clinical trials were established to assess the safety, tolerability, PKs and PDs of ASKG315 as monotherapy and in combination with pembrolizumab in individuals with advanced solid tumors (NCT05554666; NCT05509985). In addition, AskGene Pharma designed ASKG915, a tumor-selective and *cis*-potentiated anti-PD-1 antibody-IL15 bispecific therapeutic, and ASKG915 was licensed to an IND in the first quarter of 2023 (103).

In a preclinical study, Zhao et al. developed an IL-15 prodrug masked by biocompatible polymers through chemical linkers. It responds to a TME with tumor-specific high reducing potential and acidic pH value, which are features aimed at reducing systemic exposure and thus minimizing toxicity (104).

## IL-7

IL-7 is a fundamental lymphopoietic cytokine that is predominantly expressed in non-hematopoietic cells, including stromal cells, epithelial cells, endothelial cells, fibroblasts, smooth muscle cells and keratinocytes, and has been proven to be essential for the development and survival of T and B lymphocytes (105). Moreover, *IL-7* deficiency leads to severely impaired immune cell development (106). IL-7 receptor (IL-7R) is a heterodimeric complex consisting of IL-7Rα (also known as CD127) and the common cytokine receptor γ-chain, which is shared with the receptors for IL-2, IL-4, IL-7, IL-9, IL-15 and IL-21 (107). Whereas γc is expressed in most hematopoietic cells, IL-7Rα is almost exclusively expressed in cells of the lymphoid lineage, including B-cell progenitors (but not mature B cells), ILCs, naïve T cells and central memory T cells (107). Mechanistically, IL-7 crosslinks the extracellular domains of IL-7Rα and γc and promotes IL-7Rα and γc heterodimerization, leading to activation of JAK1 and JAK3, which ultimately activates major downstream signaling molecules, including STAT5 and PI3K (108) (**Figure 2A**).

As a multifunctional cytokine, IL‐7 can restore T‐cell numbers under lymphopenic conditions by boosting homeostatic cell proliferation (109). Early animal experiments consistently showed that IL-7 administered pharmacologically induced T lymphocyte proliferation while inducing little toxicity, implying that IL-7 may have a therapeutic benefit in humans. **CYT107** is a glycosylated recombinant human IL-7 (rhIL-7) developed by RevImmune (formerly Cytheris) that is well tolerated and expands the lymphocyte pool in humans after allogeneic stem cell transplantation (**Figure 2E**) (110). In a randomized placebo-controlled Phase IIa trial (NCT01362107), CYT107 significantly increased the number of CD4^+^ and CD8^+^ T lymphocytes in twenty lymphopenic metastatic breast cancer patients when administered before chemotherapy (110). Due to the small sample size, a larger clinical trial is required to manifest its impact on clinical outcomes.

Because of its low structural stability and low productivity, the commercial use of IL-7 as a therapeutic agent has been hindered by many intrinsic technical challenges. Recently, **NT-I7** (also named HyLeukin-7™; TJ107; rhIL-7-hyFc; GX-I7; and Efineptakin alfa), developed by Genexine (originating from a NeoImmuneTech-related company), which relied on its own hyFc^®^ technology platform, is a long-acting cytokine consisting of rhIL-7 fused to a hybrid Fc (hyFc) region composed of the hinge-CH2 region of immunoglobulin D (IgD) and the CH2-CH3 region of immunoglobulin G4 (IgG4) (**Figure 2E**) (111, 112). NT-I7 was engineered to serve as a stable, long-acting factor for T-cell production, maturation, amplification, trafficking, functioning and survival. Moreover, it was designed to minimize the loss of the bioactivity of drug candidates and prevent ADCC- or CDC-mediated cell killing effects. Notably, NT-I7 enhances T-cell-mediated antitumor immunity through multiple mechanisms, including expansion of the TCR repertoire; sensitization of T cells to weaker antigens; boosting naïve T cells, stem cell-like memory T cells (T_SCM_) and memory T cells; promotion of memory T-cell differentiation; enhancement of T-cell trafficking and infiltration into tumors; augmentation of the antigen-specific T-cell response; antagonization of T-cell exhaustion and other inhibitory networks; and stimulation of the proinflammatory cytokine milieu in the TME (113).

At present, NT-I7 is the only clinical-stage long-acting rhIL-7 being investigated for oncological and immunological indications, in which it promotes T lymphocyte proliferation and enhances T-cell functionality. Previous data from a Phase I study with 30 healthy volunteers (NCT02860715) showed that **NT-I7** dramatically boosted the absolute number of lymphocytes, especially T lymphocytes, and was well tolerated (114); another Phase Ib trial with 21 patients with advanced cancer (NCT03478995) showed that the drug was also well tolerated, and no DLT or cytokine release syndrome (CRS) was evident, and a dose-dependent increase in the number of lymphocytes and T lymphocytes (except Treg cells) was identified. Recently, Campian et al. demonstrated that NT-I7 mitigated RT-related lymphopenia, increased cytotoxic CD8^+^ T lymphocyte numbers throughout the body and in the tumor, and prolonged the survival of orthotopic glioma-bearing mice (111). NT-I7 is currently being evaluated for the treatment of high-grade glioblastoma in an ongoing Phase I/II trial (NCT03687957) (111). Additionally, the results from the trial confirmed that NT-I7 promoted TME inflammation by significantly upregulating the CD8^+^ T-cell to Treg cell ratio and enhanced the antitumor response by increasing the infiltration of IFN-γ-expressing T lymphocytes into the tumor. In other clinical trials, NT-I7 has exhibited favorable PKs/PDs and safety profiles when used both as a single agent and in combination with other antitumor treatments. Correspondingly, several Genexine-sponsored clinical studies of NT-I7 in combination with immune checkpoint monoclonal antibodies (namely pembrolizumab and atezolizumab) in the treatment of solid tumors including triple-negative breast cancer (TNBC), Merkel cell carcinoma (MCC), cutaneous squamous cell carcinoma (SCC) and melanoma are ongoing (NCT03752723; NCT03901573).

**MDK1319** is a small IL-7R peptidyl agonist developed by Medikine, Inc., and is fused to an IgG Fc domain to generate **MDK701** to improve *in vivo* performance (**Figure 2E**) (115). Intravenous injection of MDK-701 into cynomolgus macaques (single dose at 1 mg/kg) exhibited a circulating terminal half-life of approximately 32 hours and induced peripheral lymphocyte profiles similar to those observed after IL-7 treatment, followed by sustained elevation of peripheral lymphocyte levels, which remained higher than the baseline for 29 days with no side effects observed (115).

## IL-21

IL-21 is a pleiotropic cytokine that promotes the activation, expansion, and survival of tumor-specific CD8^+^ cytotoxic T cells while also enhancing B-cell proliferation and antibody production (116, 117). In addition, IL-21 enhances the antineoplastic activity of CD8^+^ T cells and NK cells (118, 119). IL-21 is primarily produced by CD4^+^ T and NKT cells, and the IL-21 receptor (IL-21R) is expressed on many hematopoietic cells, including CD4^+^ T follicular helper (Tfh), Treg, and T helper 17 (Th17) cells, naïve and memory CD8^+^ T cells, and B, NK, and dendritic cells (DCs) (116). IL-21R is a heterodimer consisting of a common γ-chain subunit and a specific subunit (CD360). IL-21/IL-21R engagement triggers a cascade of events that includes activation of the tyrosine kinases JAK1 and JAK3, followed by activation and phosphorylation of the transcription factors STAT3, STAT1 and STAT5 (120) (**Figure 2A**).

Early preclinical studies leveraged the overexpression of IL-21 directly in transplanted tumors or systemic administration of IL-21 plasmid DNA by hydrodynamic-mediated methods, and both of these delivery methods significantly reduced tumor growth in an NK cell- and T-cell-dependent manner (119, 121). Moreover, treatment of cynomolgus monkeys with recombinant IL-21 (rIL-21; **denenicokin**) developed by ZymoGenetics (acquired by Bristol-Myers Squibb) was associated with a dose-dependent increase in soluble CD25 (sCD25), an important marker of T-cell and NK-cell activation (**Figure 2F**) (122). A Phase I dose-elevation trial was performed, and the toxicity profile, immunomodulatory properties, and PKs of rIL-21 in patients with metastatic melanoma and RCC were recorded (122). Subsequently, in a Phase IIa clinical study, treatment with rIL-21 (30 μg/kg per dose) led to 1 CR and 1 partial response (PR) in 24 patients with metastatic melanoma. Further biomarker analyses demonstrated that rIL-21 treatment led to increased frequencies and selective activation of NK and CD8^+^ T lymphocytes, as indicated by upregulated expression of CD25, IFN-γ, perforin and granzyme B (123).

Due to the modest efficacy and short half-life of IL-21, its widespread therapeutic utility as a monotherapy has been limited. Recently, scientists from Anwita Biosciences and Shanghai Junshi Biosciences jointly engineered an IL-21-based fusion protein (**IL-21-αHSA**) in which a nanobody targeting HSA was fused to the C-terminus of rhIL-21 (**Figure 2F**) (124). αHSA nanobodies exhibited extensive species cross-reactivity and bound to an HSA epitope that does not overlap with FcRn-binding sites, prolonging the IL-21 half-life. Notably, in two syngeneic animal models, IL-21-αHSA demonstrated improved antitumor effects and exhibited synergistic antitumor benefits when combined with PD-1 and T-cell immunoreceptor with Ig and ITIM domain (TIGIT) blockers (124).

In a comparable preclinical study, Amgen Inc. developed a highly attenuated **variant of IL-21** (R9E:R76A) and fused it to an anti-PD-1 antibody, and it provided protection against a humanized mouse tumor model that was resistant to anti-PD-1 monotherapy (125). Fusion of engineered IL-21 variants to anti-PD-1 antibodies can improve the drug-like characteristics of IL-21 cytokines, thereby increasing serum cytokine half-life and allowing for less frequent dosing. Bifunctional fusion proteins can block PD-1/PD-L1 interactions while delivering the IL-21 cytokine to PD-1-positive T cells. Furthermore, Amgen Inc. sponsored a FIH Phase I trial to assess the safety, tolerability, and estimated dosing of the abovementioned agent (also known as **AMG 256**; a chimaera consisting of anti-human PD-1 variable region, a mouse IgG1 constant region and human IL-21 variant R9E:R76A monomer fused to the C-terminus) and utilized it as a single agent in patients with advanced solid tumors (NCT04362748) (**Figure 2F**) (126).

## IL-12

**Figure 3:**
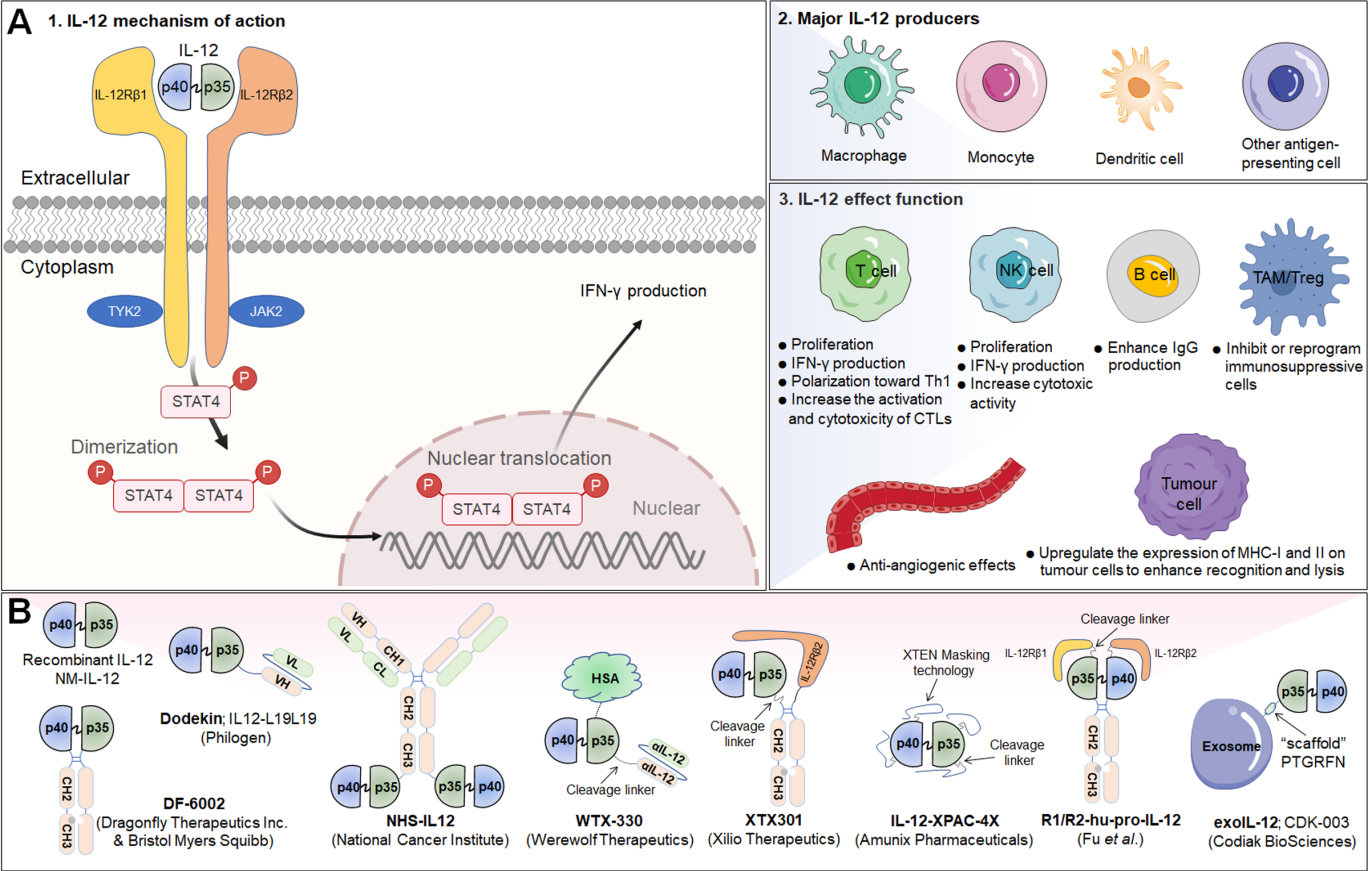
IL-12 signaling pathway, downstream effector molecules, major IL-12 producers, IL-12 effect function, and representative therapeutic candidates. **(A-1)** IL-12 is a heterodimeric cytokine consisting of IL-12p35 and IL-12p40, which bind to IL-12Rβ2 and IL-12Rβ1, respectively. IL-12 interacts with IL-12R to induce Jak2 and Tyk2 phosphorylation, followed by STAT4 phosphorylation, dimerization and nuclear translocation, ultimately promoting IFN-γ gene transcription and IFN-γ secretion. **(A-2)** Biologically active IL-12 is produced mainly by activated inflammatory cells including monocytes, macrophages, DCs, and other antigen-presenting cells. **(A-3)** Biological effects of IL-12 on different cells. **(B)** Representative therapeutic candidates for engineered IL-12.

Interleukin-12 (IL-12) is a powerful proinflammatory cytokine that has piqued the interest of researchers seeking to develop long-term cancer immunotherapy. Due to its prominent proinflammatory and immunomodulatory capabilities, IL-12 can convert immunologically suppressed “cold” tumors into inflamed “hot” tumors (127). Structurally, IL-12 is a typical heterodimeric cytokine consisting of two subunits——an α subunit (IL-12p35) and a β subunit (IL-12p40), which are linked by disulfide bonds (128). Biologically active IL-12 is produced primarily by activated inflammatory cells, including monocytes, macrophages, DCs, and other antigen-presenting cells. IL-12 activates the immune system by binding to its receptor (IL-12R), which consists of IL-12Rβ1 and IL-12Rβ2 and is predominantly expressed by activated T cells, NK cells, and DCs (128). Engagement of IL-12 with its heterodimeric receptors induces phosphorylation of JAK2 and TYK2, followed by STAT4 phosphorylation, dimerization, nuclear translocation, and IFN-γ secretion (129). Functionally speaking, IL-12 induces the differentiation of Th1 cells; increases the activation and cytotoxicity of NK and T lymphocytes; inhibits or reprograms immunosuppressive cells, such as MDSCs and tumor-associated macrophages (TAMs); upregulates the expression of MHC-I and MHC-II on tumor cells to enhance recognition and lysis; suppresses IgE production but enhances IgG production by B cells; and shows anti-angiogenic effects (130) (**Figure 3A**).

The overwhelming majority of the evidence has indicated that IL-12 activates the innate and adaptive immune systems and exerts excellent therapeutic effects by shrinking syngeneic tumors. However, the systematic administration of recombinant IL-12 (rIL-12) in clinical trials has been associated with serious immune-related side effects and a narrow therapeutic window, limiting the response rates, and no IL-12-based therapeutic drug has been approved to date. Moreover, natural IL-12 may not specifically accumulate in the TME, limiting its efficacy and potentially causing the aforementioned immune-associated adverse events. Thus, modification of IL-12 or its combination with other targeted agents may increase its efficacy and overcome safety issues (**Table 3**; **Figure 3B**).

**Table 3:** Characteristics of engineered IL-12 variants.

Name	Engineered approaches		Mechanism of actin	Tumour type	Stages	Institutions/References
mIL-12N220L	IL-12-based N-glycosylation mutant protein		Enhanced sustained cytotoxic T lymphocyte responses	Solid tumour	Preclinical	Young C. Sung et al.
DF6002 (BMS-986415)	A monovalent IL-12 immunoglobulin Fc fusion protein		Long-action; promoted sustained IFN-γ response with reduced toxicity.	Solid tumours	Phase I/II	Dragonfly Therapeutics Inc.; BMS
Mono-mIL12-Fc	Heterodimeric immunoglobulin Fc-fused mouse IL-12 (mIL-12) in a monovalent binding format		Long-action; enhanced IFN-γ secretion and the expansion of effector cells in tumours	Solid tumours	Preclinical	Yong-Sung Kim et al.
XmAb662	Potency-reduced IL12-Fc fusion protein		Increased NK and T lymphocytes number, serum IP10 and IFN-γ production	Advanced solid tumours	Planned IND application in 2023	Xencor Inc.
Dodekin (IL12-L19L19)	Immunocytokine; a vascular targeting antibody fused to IL-12		Tumour-targeted and immune activation	Advanced solid carcinomas; DLBCL	Phase I/II	Philogen S.p.A. and Luciano Zardi et al.
IL12-F8-F8	Immunocytokine; scFv(F8) specific to EDA of fibronectin fused to IL-12		Tumour-targeted and immune activation	Lymphoma; Solid tumours	Preclinical	Nadine Pasche et al.
NHS-IL12	Immunocytokine; two IL-12p70 heterodimers fused to each CH3 C-terminus of human monoclonal IgG1 antibody NHS76		Necrosis-targeted and immune activation	Solid tumours	Phase I/II	National Cancer Institute (NCI)
CBD-IL-12	Immunocytokine; collagen-binding molecular fused to IL-12		Induced sustained intratumoural levels of IFN-γ	Breast cancer	Preclinical	Aslan Mansurov et al.
Masked IL-12	IL-12-prodrug; fused a domain of the IL-12R to IL-12 via a protease-cleavable linker		Tumour-selective release; rendered cold tumours responsive	Solid tumours	Preclinical	Aslan Mansurov et al.
WTX-330	Conditionally activated IL-12 prodrug; an HSA and wild-type IL-12 masked with anti-IL-12 scFv via tumour protease-sensitive linkers		Long-action; tumour-selective protease activation; amplified antitumour T effector cell responses	Solid tumours	Planned IND application in 2023	Werewolf Therapeutics
XTX301	Engineered IL-12 prodrug; Knob&hole hFc; Masking with IL-12Rβ2 moiety		Long-action; tumour-selective protease activation; improved IFN-γ production	Solid tumours	IND application accepted in 2022	Xilio Therapeutics
R1/R2-hu-pro-IL-12	Tumour-conditional IL-12 prodrug; using ECD from both human IL-12Rβ1 and IL-12Rβ2 to mask IL-12 activity		Long-action; tumour-selective matrix metalloproteases activation; preferentially and persistently activating TILs	Solid tumours	Preclinical	Yang-Xin Fu et al.
IL-12-XPAC-4X	Protease-activated IL-12 prodrug; XPAC™ platform; XTEN^®^ polypeptide-fused protease-activated cytokines		Long-action; tumour-selective protease activation; immune stimulation	Solid tumours	Preclinical	Amunix Pharmaceuticals
IL-12 prodrug (Unnamed)	Tumour-selective IL-12 prodrug; Azymetric™ Fc technology; Single or double anti-IL-12 scFv was fused by a protease-cleavable linker to mask IL-12 activity		Long-action; tumour-selective protease activation; immune stimulation	Solid tumours	Preclinical	Zymeworks
Alum-tethered IL-12	Recombinant IL-12 bound tightly to the vaccine adjuvant alum		Vaccine adjuvant; long retention in the tumours; substantial IFN-γ-mediated T-cell and NK-cell activities	Melanoma tumours	Preclinical	K. Dane Wittrup et al.
exoIL-12	IL-12 fused with PTGFRN (as a scaffold) preformed in exosome		Long-action; improved therapeutic window	Early stage cutaneous T-cell lymphoma	Phase I	Codiak BioSciences
IL-12 INS-CAR T	IL-12-loaded HSA nanoparticles are efficiently conjugated onto CAR T cells		Immune-boosting effects of IL-12 enhance CAR T-cell antitumour abilities	Solid tumours	Preclinical	Luo et al. (149)

In a prior report, Young C. Sung and team engineered an IL-12-based N-glycosylation mutant (**mIL-12N220L**) that increases durable cytotoxic T-cell immune responses and induces more effective protection against tumor challenge than wild-type IL-12. Thus, this mutant IL-12 was employed to develop DNA vaccines that can be used as an adjuvant for the production of long-term memory T-cell responses (131).

To further improve the half-life of IL-12 *in vivo* and broaden the therapeutic window, several fusion proteins of IL-12 and immunoglobulin Fc with comparable structures were applied for clinical development. For example, a preclinical research team from Ajou University School of Medicine, developed heterodimeric immunoglobulin Fc-fused mouse IL-12 (mIL-12) in a monovalent-binding format (**Mono-mIL12-Fc**) to produce long-acting mIL-12 in the natural heterodimeric form. Mono-mIL12-Fc displayed a much longer plasma half-life than recombinant mIL-12 and can be administered systemically twice weekly, which eliminated established tumors in syngeneic mouse models without inducing noticeable toxicity. More importantly, mono-mIL12-Fc elicited weak IL-12 signaling, favoring the generation of functional and protective memory CD8^+^ T lymphocytes (132).

Subsequently, Dragonfly Therapeutics Inc. and BMS jointly developed **DF-6002** (BMS-986415), a monovalent IL-12-immunoglobulin Fc fusion protein, as an experimental immunotherapy for the treatment of advanced solid tumors in adult patients. A Phase I/II trial established to evaluate DF6002 administered alone and in combination with nivolumab in participants with locally advanced or metastatic solid tumors is ongoing (NCT04423029) (133).

Another clinically investigated potency-reduced IL-12-Fc fusion protein is **XmAb662**, which is aimed at increasing tumor immunogenicity. XmAb662 is being further studied and, to date, has demonstrated significant antitumor activity *in vivo*, and has shown the ability to increase the numbers of NK cells and T lymphocytes and the levels of interferon gamma-induced protein 10 (IP-10) and IFN-γ in serum. Moreover, these effects were enhanced when XmAb662 was combined with an anti-PD-1 antibody. In 2023, Xencor Inc., a biopharmaceutical company that develops clinical-stage drugs, plans to submit an IND application for XmAb662 and initiate a Phase I trial for participants with advanced solid tumors.

### IL-12-based immunocytokines

Considering that immune cells in the tumor and surrounding draining lymph nodes are ideal targets for IL-12 immunotherapy, there have been various initiatives to develop tumor-targeted IL-12 therapeutics (**Table 3**; **Figure 3B**). In an earlier study, Verena Gafner et al. developed three structurally similar IL-12-based fusion proteins (**IL12-scFv(L19)-FLAG**) based on the sequential fusion of a single-chain IL-12 derivative to an anti-EDB antibody fragment scFv (scFv(L19)) (134). One of three candidate compounds, in which p40 and p35 formed a covalent heterodimer and each subunit was fused to a molecule of scFv(L19) (known as **p40-scFv(L19)/scFv(L19)-p35**), demonstrated excellent tumor-targeting efficacy, as evaluated via biodistribution analysis, and potent antitumor activity in animal models of teratocarcinoma, showing superior performance compared to that of the other two IL12-scFv(L19)-FLAG candidates (134). Based on the aforementioned study, Philogen developed **Dodekin** (IL12-L19L19), a fully human immunostimulatory candidate consisting of a vasculature-targeting antibody fused to IL-12, a tumor-targeted product that selectively localizes to the site of tumor lesions while safeguarding healthy tissues (135). Considering encouraging preclinical data, Philogen has initiated a Phase I clinical study to assess the safety and early signs of efficacy of Dodekin in patients with advanced solid carcinomas and DLBCL that have progressed after immune-checkpoint blockade therapy (NCT04471987).

**NHS-IL12** is another necrosis factor-targeting immunocytokine consisting of two IL-12p70 heterodimers in which each CH3 C-terminus is fused to the anti-human monoclonal IgG1 antibody NHS76, capitalizing on the specific affinity of NHS76 antibody for ssDNA and dsDNA, which are frequently exposed in the necrotic portions of tumors (136). Because human IL-12 does not cross-react with murine IL-12R, a chimeric surrogate immunocytokine, NHS-muIL12, was constructed and tested in preclinical experiments with immunocompetent mice. Systemic administration of the murine analogue NHS-muIL12 delayed the growth of MC38-CEA^+^ colorectal carcinomas in carcinoembryonic antigen transgenic (CEA.Tg) mice (137). Currently, NHS-IL12 is being investigated in Phase I/II clinical trials as a single agent (NCT01417546) or in combination with M7824 (an anti-PD-L1/TGFβ trap fusion protein) (NCT04303117) in solid tumors. Interim data showed that NHS-IL12 had good tolerability with an MTD of 16.8 µg/kg, with 9% of cancer patients experiencing disease stability (138).

Recently, Aslan Mansurov and his coworkers created a novel fusion protein by fusing collagen-binding molecule to IL-12, designated **CBD-IL-12**, which can target tumor lesions and accumulate in the tumor stroma, where collagen is exposed due to a disorder in the tumor vascular system. *In vivo* data illustrated that intravenous administration of CBD-IL-12 stimulated sustained IFN-γ levels within tumors, significantly reduced IFN-γ levels in the peripheral circulatory system, attenuated organ damage and exhibited excellent anticancer activity, triggering complete remission of checkpoint inhibitor-resistant breast tumors (139).

### IL-12-based prodrugs

Notably, there are several structurally distinct IL-12 prodrugs in clinical development designed to further improve the safety of treatment (**Table 3**; **Figure 3B**). **WTX-330** is a systemically administered, selectively activated IL-12 prodrug consisting of an HSA to extend its half-life and wild-type IL-12 masked with a high-affinity anti-IL-12 scFv that are connected via tumor protease-sensitive linkers (140, 141). WTX-330 was developed to include a high-affinity blockade of IL-12/IL-12R engagement systemically via the circulatory system and in nontumor tissues to prolong the half-life of IL-12 for optimal tumor exposure and tumor-selective protease activation through a proprietary mechanism. In preclinical investigations, WTX-330 exhibited prominent antitumor potential, as well as a favorable PK and tolerability profile. Robust antitumor activity was mediated through stimulation of innate and adaptive antitumor responses, including DC maturation and cross-presentation of antigens, Th1 cell differentiation, and expansion of antitumor effector T lymphocyte responses. Werewolf Therapeutics filed an IND for WTX-330 in the third quarter of 2022.

**XTX301** is a protein-engineered IL-12 prodrug that is masked with a protein domain to block binding activity until IL-12 is cleaved by TME-related proteases. To assess tumor-selective activity, antitumor potency and peripheral toxicities, a murine surrogate (mXTX301) was constructed. Preclinical results showed that intravenous administration of mXTX301 at a single dose of 0.5 mg/kg inhibited tumor growth by up to 90% in MC38 (inflamed) and B16F10 (noninflamed) syngeneic mouse models. Moreover, mXTX301 induced an approximately 3-fold increase in the IFN-γ levels within the tumor compared to the vehicle control and an approximately 150-fold reduction in peripheral IFN-γ levels compared to mXTX300 (the unmasked control). More importantly, XTX301 was well tolerated and activated in a protease-dependent manner. Xilio Therapeutics is progressing XTX301 through IND-enabling studies and plans to investigate XTX301 as a single agent and combination therapy for the treatment of solid tumors (142).

Recently, in a preclinical study, Professor Yang-Xin Fu and team generated a conditional IL-12 prodrug (**Pro-IL-12**; R1/R2-hu-pro-IL-12) composed of IL-12 masked by IL-12 extracellular receptor-binding domains consisting of both human IL12Rβ1 and IL12Rβ2 that can be released by cleavage of an MMP linker, which is preferentially activated inside the TME and shows limited systemic toxicity. The results showed that the activity of this pro-IL-12 shielded IL-12 compound *in vitro*, showed reduced systemic toxicity *in vivo*, and effectively controlled both primary and metastatic tumor growth in MC38, B16-F10, and 4T1 mouse tumor models. Importantly, Pro-IL-12 used in combination with an anti-PD-L1 antibody and a tyrosine kinase inhibitor (TKI) induced robust tumor regression in a TUBO model, a representative model of cold tumors, and an MC38 model (143).

Notably, Amunix Pharmaceuticals (acquired by Sanofi in 2021) recently applied its proprietary masking technology (the XPAC™ platform; XTEN^®^ polypeptide-fused protease-activated cytokines) to its first masked protease-activated cytokine platform, **IL-12-XPAC-4X (** 29). Concurrently, Zymeworks engineered **another novel IL-12-prodrug** (unnamed to date) in which single-chain IL-12 was fused to one C-terminus of Zymeworks’ Azymetric™ Fc heterodimer (144). An anti-IL-12 scFv or anti-p35 scFv was fused to the other Fc heterodimer C-terminus via a protease-cleavable linker to block IL-12 activity. Leveraging linkers engineered to be cleaved by highly active intratumoral proteases, blocking antibodies are released selectively within the TME, hence boosting intratumoral IL-12 activity. Comprehensive published data on these two IL-12-based prodrugs are currently unavailable.

### Other IL-12-based drugs

Alum has been safely used in humans for approximately 100 years and has been approved by the FDA as a vaccine adjuvant (145). Alum adjuvants consist of micrometer-sized (1-10 mm) aggregates of rod-shaped aluminum oxyhydroxide nanocrystals, which form a physical deposit at injection sites in tissue that persists for weeks. Interestingly, researchers from MIT constructed an engineered **alum-tethered IL-12** in which recombinant IL-12 was bound tightly to the vaccine adjuvant alum via ligand exchange between hydroxyls on the surface of alum and phosphoserine residues attached to the cytokine by an alum-binding peptide. Specifically, the *in vivo* results demonstrated that in a murine melanoma model, the intratumoral infusion of alum-tethered IL-12 exhibited retention for weeks in the tumors along and induced minimal side effects while inducing substantial IFN-γ-mediated NK and T lymphocyte activity, thus increasing tumor antigen accumulation in draining lymph nodes and triggering strong tumor-specific T-cell priming (146).

Exosomes are natural, cell-derived vesicles that compose a nonimmunogenic delivery system for a variety of therapeutic payloads (147). Codiak BioSciences recently generated a novel engineered IL-12-based exosome therapeutic candidate (**exoIL-12**; CDK-003) by displaying functional IL-12 on the exosome surface by fusing the abundant exosomal surface protein PTGFRN as a “scaffold” (148). ExoIL-12 was designed to address the limitations of undesired systemic exposure and unpredictable pharmacology. In preclinical studies, exoIL-12 demonstrated longer tumor retention and better antitumor effects than wild-type IL-12 following intratumoral injection, thereby creating a therapeutic window for this powerful cytokine (148). Codiak BioSciences has initiated a Phase I/IIa clinical study for exoIL-12 with healthy volunteers (subcutaneous administration) and patients with early stage cutaneous T-cell lymphoma (intralesional administration) (NCT05156229).

Chimeric antigen receptor T (CAR T) cell immunotherapy has shown significant success in the treatment of hematological tumors, but its therapeutic benefits are greatly limited in solid tumors due to the lack of activated T lymphocytes effectively infiltrating the immunosuppressed TME. Recently, Luo et al. designed an IL-12-based nanostimulant (INS; as a nanochaperone)-functionalized CAR T-cell (**INS-CAR T**) biohybrid, in which IL-12-loaded HSA nanoparticles were efficiently conjugated with CAR T cells via biorthogonal chemistry without affecting the antitumor potential of these engineered cells. IL-12 is reactively released from INS-CAR T-cell biohybrids after the number of tumor antigen-triggered thiol groups is increased. Finally, the immune-boosting effects of the IL-12 nanochaperone greatly enhanced CAR T-cell antitumor activity, markedly eradicated solid tumors and reduced undesired adverse events (149).

## IL-18

**Figure 4:**
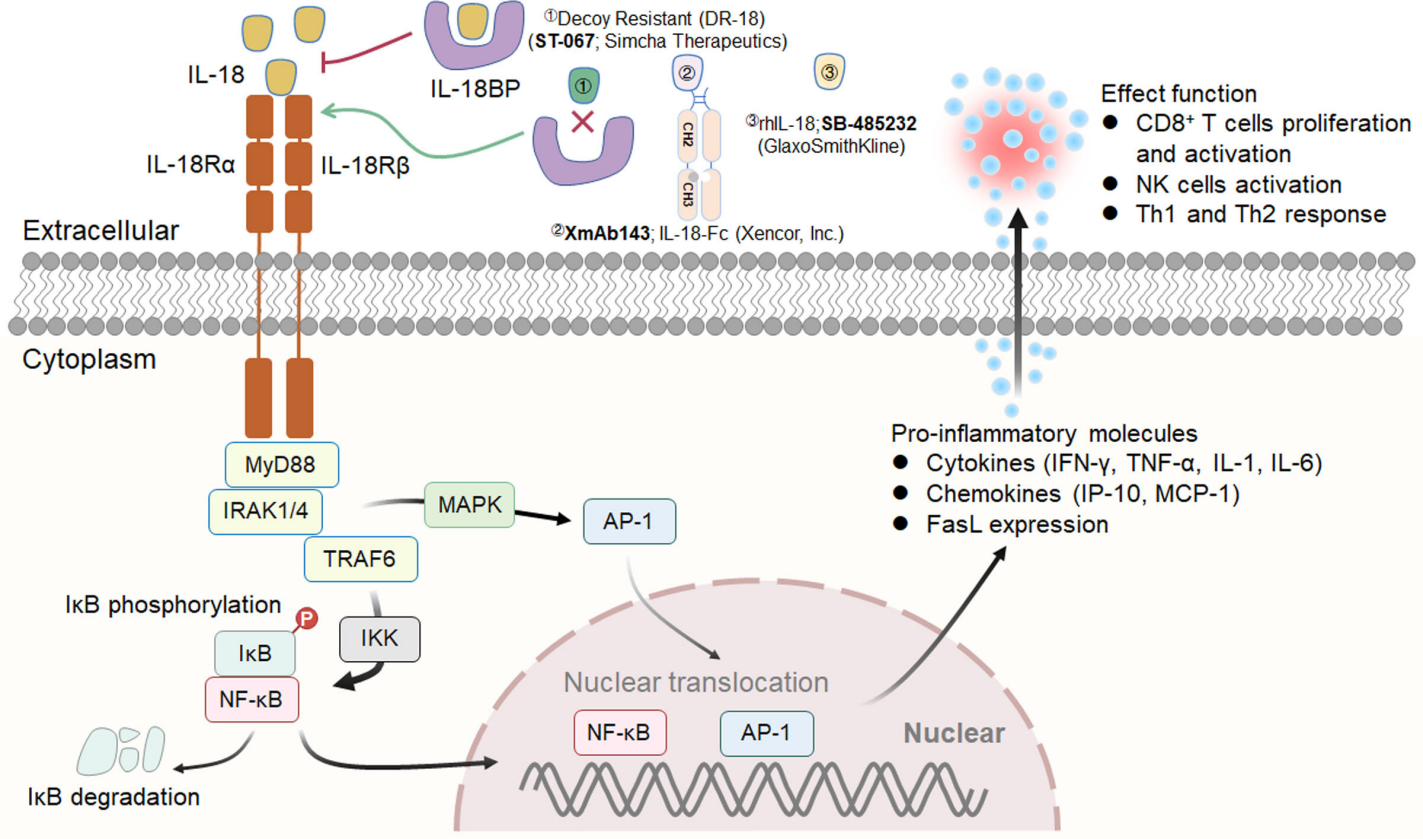
IL-18 signaling pathway, downstream effector molecules and representative therapeutic candidates. IL-18 drives the MyD88/NF-κB and MAPK signaling pathways through heterodimerization of its receptor subunits IL-18Rα and IL-18Rβ. Three representative therapeutic candidates of engineered IL-18 are shown (*above*).

Interleukin (IL)-18, formerly known as interferon gamma-inducing factor (IGIF), belongs to the IL-1 cytokine family and mediates inflammation downstream of activated NLRP3 and NLRP1 inflammasomes. IL-18 is abundant in macrophages, DCs and epithelial cells in the form of an inactive precursor (pro-IL-18) (150). When inflammation is triggered, pro-IL-18 is cleaved by caspase-1, a reconstituted NLRP3 inflammasome component, becoming active IL-18 and is rapidly released (151). Mechanistically, IL-18 drives the MyD88/NF-κB and MAPK signaling pathways through heterodimerization of its receptor subunits IL-18Rα (IL18R1; CD218a) and IL-18Rβ (IL18RAP; CD218b) (152). IL-18R has been shown to be expressed on normal peripheral blood lymphocytes (153). Increasing evidence indicates that IL-18 not only strongly induces the production of cytokines such as IFN-γ and TNF-α from immune cells but also increases the expression of FasL and enhances the toxicity of NK cells (154) (**Figure 4**).

In a preclinical animal study, Ma et al. investigated the effect of recombinant mouse IL-18 (provided by GlaxoSmithKline) on the peritoneal dissemination of CT-26 cells or tail vein infusion of metastatic B16/F10 cells in combination with an anti-PD-L1 antibody and/or anti-CTLA-4 antibody (155). The experimental data illuminated that IL-18 boosted the therapeutic efficacy of immunological checkpoint blockade by mediating the accumulation of premature NK cells and memory-type CD8^+^ T lymphocytes while suppressing Treg cells (155). Thus, IL-18 in combination with immune checkpoint inhibitors may be a promising strategy for developing next-generation cancer immunotherapy.

An early Phase I, dose-escalation trial documented the toxicity, PKs, and biological activities of recombinant human IL-18 (rhIL-18; **SB-485232**, developed by GlaxoSmithKline) in participants with advanced solid tumors and lymphomas, and the data demonstrated that rhIL-18 administration was safe and that the agent was well tolerated (**Figure 4**) (156). Subsequently, a Phase II trial was planned to assess the antitumor effect of three dose groups of rhIL-18 (0.01, 0.1, and 1.0 mg/kg/day) delivered as monotherapy in patients with previously untreated metastatic melanoma (157). Unfortunately, rhIL-18 failed to show efficacy in clinical studies, and none of the three doses investigated were recommended for Phase III valuation.

In a recent study, Professor Aaron M. Ring and colleagues revealed that IL-18-binding protein (IL-18BP), a secreted high-affinity (Kd<1 nM) IL-18 decoy receptor, is often upregulated in a variety of human and murine tumors and is a barrier to the antitumor activity of IL-18 (158). Subsequently, these authors engineered a variant of decoy-resistant IL-18 (**DR-18**) that does not bind the decoy receptor IL-18BP and retains the ability to bind tumor-infiltrating lymphocytes. Surprisingly, injecting DR-18 into tumor-bearing animals triggered the selective proliferation of stem-like TCF1^+^CD8^+^ T lymphocytes in tumors, as well as preferential differentiation towards polyfunctional T lymphocytes rather than an exhaustion phenotype. Additionally, DR-18 stimulated the expansion of NK cells in MHC class I-deficient tumors, suggesting a potential treatment option for immune checkpoint blockade therapy-unresponsive tumors. Simcha Therapeutics is currently sponsoring a Phase I/II clinical study (NCT04787042) designed to evaluate the safety of DR-18 (**ST-067**) as a single agent administered subcutaneously weekly for a variety of cancer indications, including melanoma, RCC, TNBC and NSCLC (**Figure 4**). Additional results of the Phase II trial, which is expected to be completed in 2023, will include PKs/PDs, clinical activity, and safety data (159).

Another IL-18 fusion protein investigated is **XmAb143** developed by Xencor, which is an engineered IL18 heterodimer fc fusion protein with improved stability, reduced potency, and insensitivity to IL18BP (**Figure 4**). In a preclinical study, XmAb143 (IL18-Fc) promoted NK and T lymphocyte expansion and IFN-γ secretion in huPBMC-engrafted NSG mice (GvHD model) and demonstrated tumor growth inhibition, T-cell proliferation and activation in CD34^+^ humanized mice (160).

## IL-10

**Figure 5:**
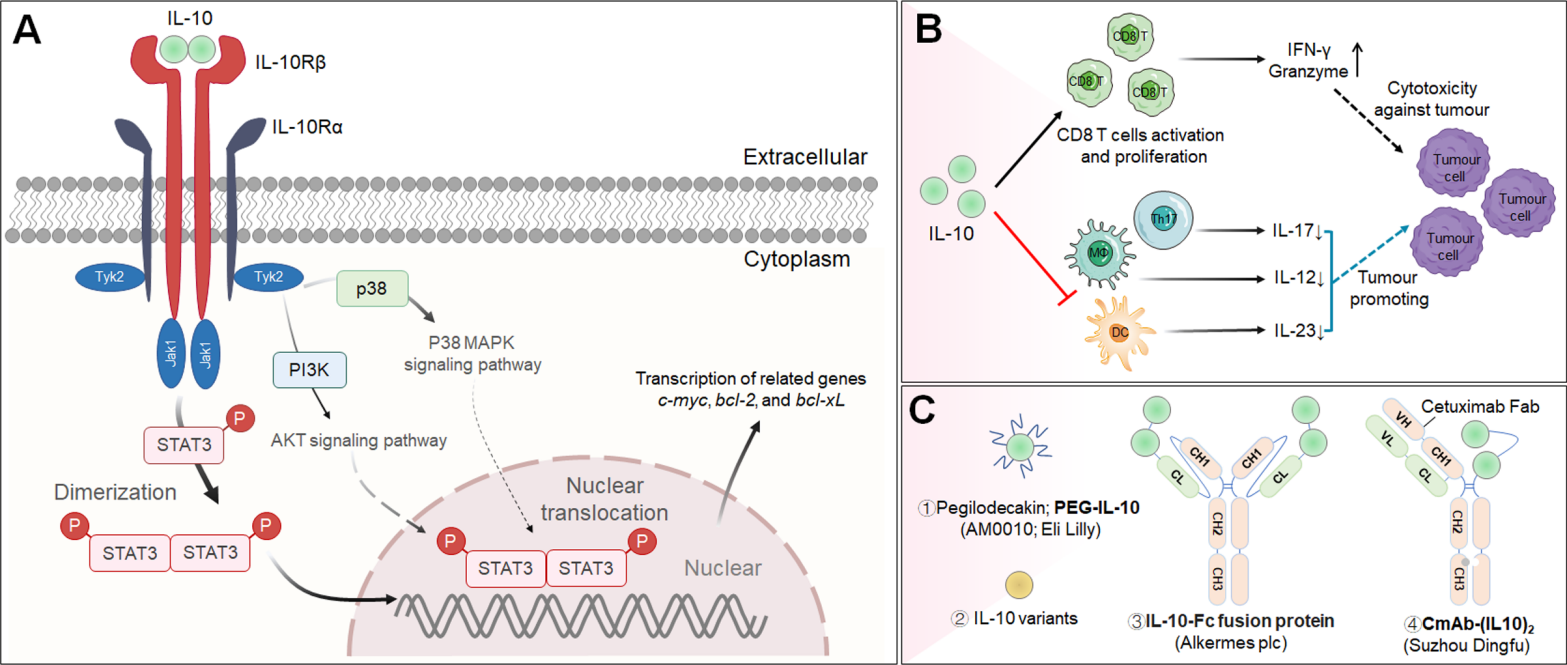
The cascade of the IL-10 signaling pathway and its downstream effector molecules and representative therapeutic candidates. **(A)** IL-10 signaling pathway and downstream cellular effects. **(B)** Immunopromoting and immunosuppresive mechanisms of IL-10 in cancer immunotherapy. **(C)** Representative therapeutic candidates of engineered IL-10.

IL-10 is a soluble homodimer cytokine that is generally considered an immunosuppressive mediator due to its prominent anti-inflammatory effects, which ensure host protection from excessive responses to pathogens and microorganisms (161, 162). Genetic ablation of *Il10* demonstrated the critical role of *Il10* in regulating inflammation, as IL-10-deficient mice spontaneously developed colitis (163). IL-10 can be produced by most T cells (including Treg cells), DCs, macrophages, epithelial cells and tumor cells (164). Mechanistically, IL-10 exerts its effects after binding to a surface IL-10 receptor (IL-10R), which is a tetramer composed of two α subunits (IL-10Rα) and two β subunits (IL-10Rβ); the α subunit is expressed on hematopoietic cells, while the β subunit is expressed ubiquitously (165). IL-10 has a strong affinity for IL-10Rα but a substantially weaker affinity for IL-10Rβ (166). IL-10 induces the dimerization of IL-10Rα and IL-10Rβ, resulting in the phosphorylation and activation of the JAK1/TYK2/STAT3/STAT1 signaling pathway and the induction of IL-10-related gene expression, including *c-myc*, *bcl-2*, and *bcl-xL* (167) (**Figure 5A**).

Recently, increasing evidence has proven that IL-10 induces antitumor activity increasing CD8^+^ T lymphocyte activation in immunologically “cold” cancers (168, 169). For example, Mumm et al. suggested that IL-10 participates in effective antitumor immune surveillance through several basic mechanisms, including by increasing the count of intratumoral tumor-specific cytotoxic CD8^+^ T lymphocytes, promoting the secretion of the cytokine IFN-γ from Th1 cells and granzyme from CD8^+^ T lymphocytes, improving the activity antigen presentation machinery and thus inhibiting tumor growth (**Figure 5B**) (170). Although the infusion of IL-10 has been identified as a potential strategy for cancer immunotherapy, its long-term therapeutic efficacy has been hampered partially because it is rapidly cleared from the circulatory system.

**Pegilodecakin** (PEGylated IL-10; PEG-IL-10; AM0010) is an IL-10-based antineoplastic agent that stimulates systemic immune responses and enhances intratumoral CD8^+^ T lymphocyte invigoration in cancer patients, according to early preclinical studies (**Figure 5C**) (171). Subsequently, a Phase I clinical study elucidated that pegilodecakin exhibits an acceptable toxicity profile and shows promising antitumor effects in the treatment of participants with advanced solid tumors (NCT02009449) (172). Despite these encouraging preclinical and Phase I clinical results, pegilodecakin in combination with FOLFOX chemotherapy (folinic acid, 5-fluorouracil and oxaliplatin) for metastatic pancreatic cancer failed to prolong OS in a Phase III clinical study (NCT02923921) (173). In addition, two Phase II clinical trials of pegilodecakin combined with immune checkpoint monoclonal antibodies (pembrolizumab or nivolumab) for metastatic NSCLC failed to meet primary endpoint criteria (NCT03382899 and NCT03382912) (174). Because the rationale for using pegilodecakin is based on strong scientific evidence, the reasons for its clinical failure require more research, including studies on the dose, activity, and concentration that is trafficked to tumors. Indeed, an early preclinical study revealed that untargeted IL-10 treatment poses substantial risks for generating toxicity by inducing CD8^+^ T lymphocyte infiltration in healthy tissue, possibly due to off-tumor delivery of PEG-IL-10 (170).

Interestingly, Gorby et al. developed multiple **IL-10 variants** with different affinities for IL-10Rβ that can recruit IL-10Rβ more efficiently than the wild type to activate cellular surface signaling complexes and trigger STAT1 and STAT3 activation at a greater rate in monocytes and CD8^+^ T lymphocytes, potentially opening up new avenues for IL-10-based antitumor immunotherapy (**Figure 5C**). Furthermore, this study shed light on how IL-10R complex stability fine-tunes IL-10 biology and suggested new possibilities for reviving failed IL-10 therapies (175).

T-cell exhaustion is a crucial obstacle to effective cancer therapy. Guo et al. engineered an **IL-10-Fc fusion** protein with a longer half-life than previous IL-10-based drugs that directly and potently increased the proliferation and effector function of terminally exhausted tumor-infiltrating CD8^+^ T cells by enhancing oxidative phosphorylation, a process that was triggered independent of exhausted progenitor T cells (**Figure 5C**). When combined with adoptive T-cell transfer immunotherapy, IL-10-Fc was discovered to be a safe and highly effective metabolic intervention to eradicate well-established solid tumors and provide long-lasting treatment for most treated mice. These results testified that metabolic reprogramming through activation of mitochondrial pyruvate carrier-dependent oxidative phosphorylation can revive terminally exhausted T lymphocytes and improve the responsiveness to cancer treatment (176).

More recently, Professor Fu and team developed a cetuximab-based IL-10 immunocytokine (**CmAb-(IL10)_2_**) for EGFR-targeted delivery of IL-10 to tumor locations in conjunction with prolonged PKs and reduced toxicity via IL-10 regulation of IFN-γ production in DCs and enhanced CD8^+^ T lymphocyte-dependent antitumor responsiveness (**Figure 5C**). The fusion protein showed great effectiveness when administered alone or combined with anti-PD-L1/CTLA-4 antibody. Structurally, CmAb-(IL10)_2_ is a bispecific Fc fusion protein that links the Fab fragment of cetuximab and an IL-10 dimer, respectively (177). At present, the National Medical Products Administration (NMPA) of China accepted Suzhou Dingfu Target Biotechnology’s clinical application of an anti-EGFR antibody/IL-10 fusion protein in 2020.

## Interferons

**Figure 6:**
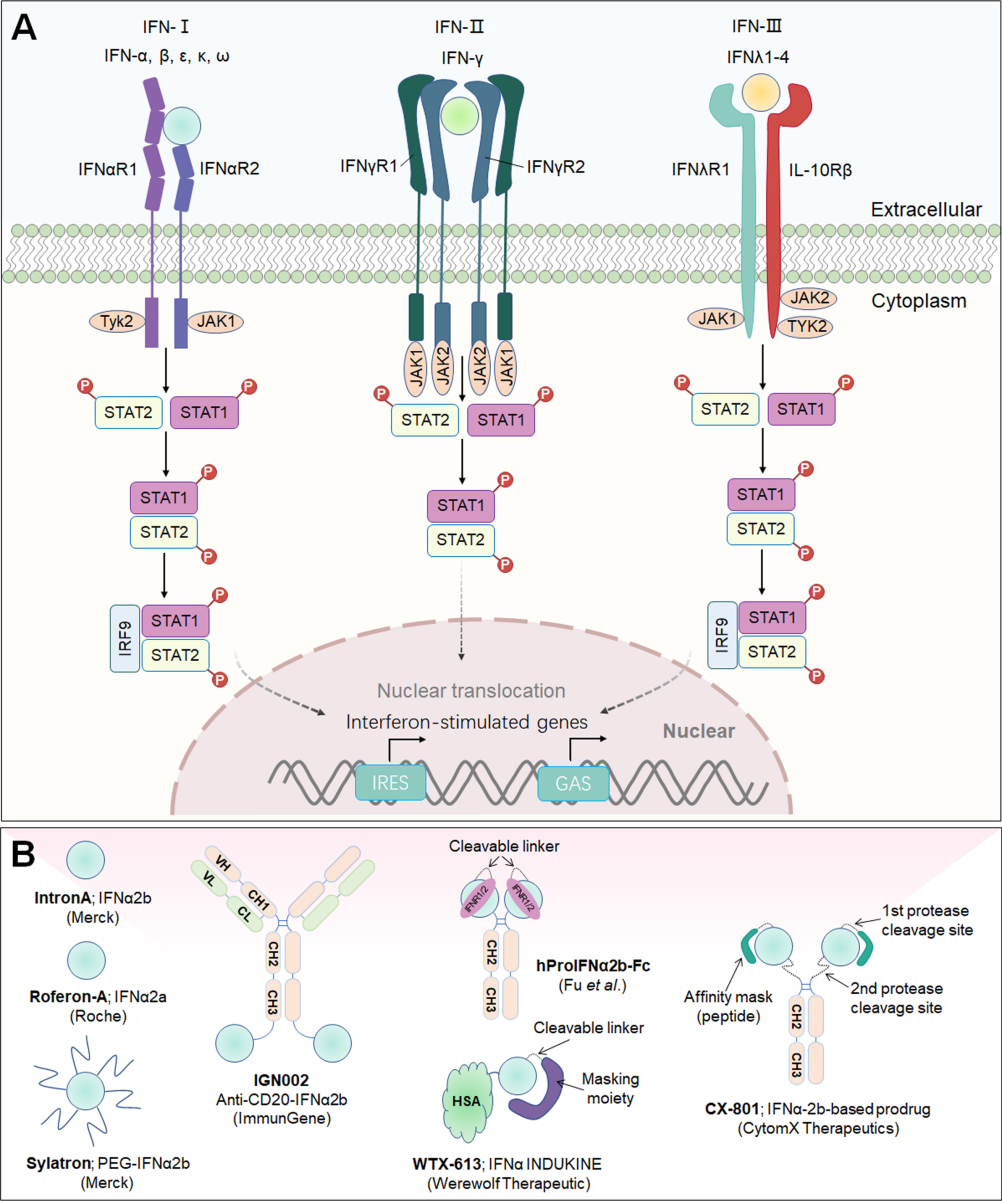
Interferon signaling pathway and downstream effector molecules and representative therapeutic candidates. **(A)** Type-I, type-II and type-III interferons, their receptors and signaling pathways. Engagement of IFNs with their corresponding receptors triggers signal transduction, mainly through the JAK/STAT pathway, followed by the phosphorylation and nuclear translocation of STAT1 and STAT2 and the activation of a large panel of ISGs. **(B)** Representative therapeutic candidates of engineered IFNs.

Interferons (IFNs) constitute a vast family of α-helical cytokines that were first discovered because of their powerful antiviral properties. IFNs are now acknowledged for their capacity to have a wide range of cellular effects, from direct cytotoxicity to immunological modulation. According to an IFN sequence identity and engagement with distinct cell surface receptors, three major types of IFNs have been classified: Type-I IFNs (IFN-α, IFN-β, IFN-ϵ, IFN-κ and IFN-ω), Type-II IFN (IFN-γ), and Type-III IFN (IFN-λ), and their receptors are IFNαR1/IFNαR2, IFNγR1/IFNγR2, IFNλR1/IL1-Rβ, respectively (178). IFNs interact with their cognate receptors to initiate signal transduction, mainly through the JAK/STAT pathway, followed by the phosphorylation and nuclear translocation of STAT1 and STAT2, and to activate a large panel of interferon-stimulated genes (ISGs) that regulate innate and adaptive immune responses as well as antiviral and antitumor immunity (179) (**Figure 6A**).

Interferons have been approved anticancer therapies, but their use has been limited by narrow therapeutic windows. Several IFNα2 alleles have been described. The best characterized are IFNα2a and IFNα2b, both of which have been marketed for clinical use as Roferon A and Intron A, respectively. Intron-A^®^ (recombinant interferon alfa-2b from Merck) was the first agent approved for adjuvant therapy for melanoma patients with a high risk for recurrence after surgical resection (**Figure 6B**) (180). Roferon-A^®^ (recombinant interferon alfa-2a from Roche) is authorized for the treatment of chronic hepatitis C and hairy cell leukemia in patients aged 18 years and older (**Figure 6B**) (181).

**Sylatron™** (an agent launched by the Schering Corporation, a subsidiary of Merck & Co., Inc.) is a covalent coupling of recombinant IFNα-2b with monomethoxy PEG that is approved for the adjuvant therapy of melanoma patients with microscopic or gross nodal involvement within 84 days after surgical resection, including complete lymph node dissection (**Figure 6B**) (182).

**IGN002** is a novel recombinant immunocytokine composed of an anti-CD20 antibody (rituximab) fused to human IFNα-2b via a peptide linker developed by ImmunGene (**Figure 6B**). A Phase I study of IGN002 is currently underway to evaluate its efficacy in patients with refractory NHL (NCT02519270) (183).

**CX-801**, designed by CytomX Therapeutics, is a wholly owned IFNα-2b-based prodrug based on Probody™ technology (**Figure 6B**). According to reports of preclinical results, CX-801 revealed a broad therapeutic scope with an improved tolerability profile when compared to unmasked IFN without compromised antitumor effects (184, 185). CX-801 has a broad range of potential applications in both traditionally immuno-oncology-sensitive (hot) and immuno-oncology-insensitive (cold) tumors. An IND submission is planned for 2023.

**WTX-613** (licensed by Jazz Pharmaceuticals) is a systemically administered, selectively activated IFNα molecule based on INDUKINE™ approaches and is being developed to lessen the serious toxic side effects associated with IFNα monotherapy while maximizing clinical benefit when used as a single agent or in combination with checkpoint antibodies in patients with refractory and/or immunologically unsensitive tumors (**Figure 6B**) (186). This drug candidate was designed to induce an efficient blockade of IFNα/IFNR engagement in the circulatory system and nontumor tissues, extending the half-life for optimal tumor exposure and proprietary tumor-selective protease activation. In experimental animal models, WTX-613 exhibited potent antitumor efficacy mediated through the induction of a type I interferon immune response with favorable PKs and tolerability profiles (186, 187).

Recently, Professor Yang-Xin Fu and team engineered a receptor-masked IFNα-2b prodrug (**hProIFNα2b-Fc**; ProIFN) in which components are connected by a linker that can be cleaved by tumor-related proteases (**Figure 6B**). hProIFNα2b-Fc showed an extended serum half-life, reduced systemic toxicity, and a long-term tumor-targeting ability compared to the unmasked form. Strikingly, ProIFN-challenged mice exhibited enhanced DC cross-priming and significantly boosted CD8^+^ T lymphocyte infiltration and effector function within the TME. ProIFN also demonstrated synergistic effects with checkpoint blockade in established tumors, as well as radiotherapy efficacy in both primary and metastatic tumors, along with superior long-term PKs with minimal toxicity in nonhuman primates (188).

Notably, the abundant expression of the IFN-γ receptor on almost all tissues limits the use of IFN-γ as a therapeutic approach. While current evidence has clarified that it is possible to leverage the immunostimulatory ability of IFN-γ, early clinical data showed inconsistent outcomes with this cytokine, which could be partially attributed to immune checkpoint receptor upregulation (i.e., PD-L1) antagonizing the immunostimulatory effects. As a consequence, IFN-γ is not approved by the FDA for the treatment of cancers thus far, although it remains a promising candidate in the modern era of immunotherapy, in which the use of checkpoint blockades is increasing.

## Tumor necrosis factor

**Figure 7:**
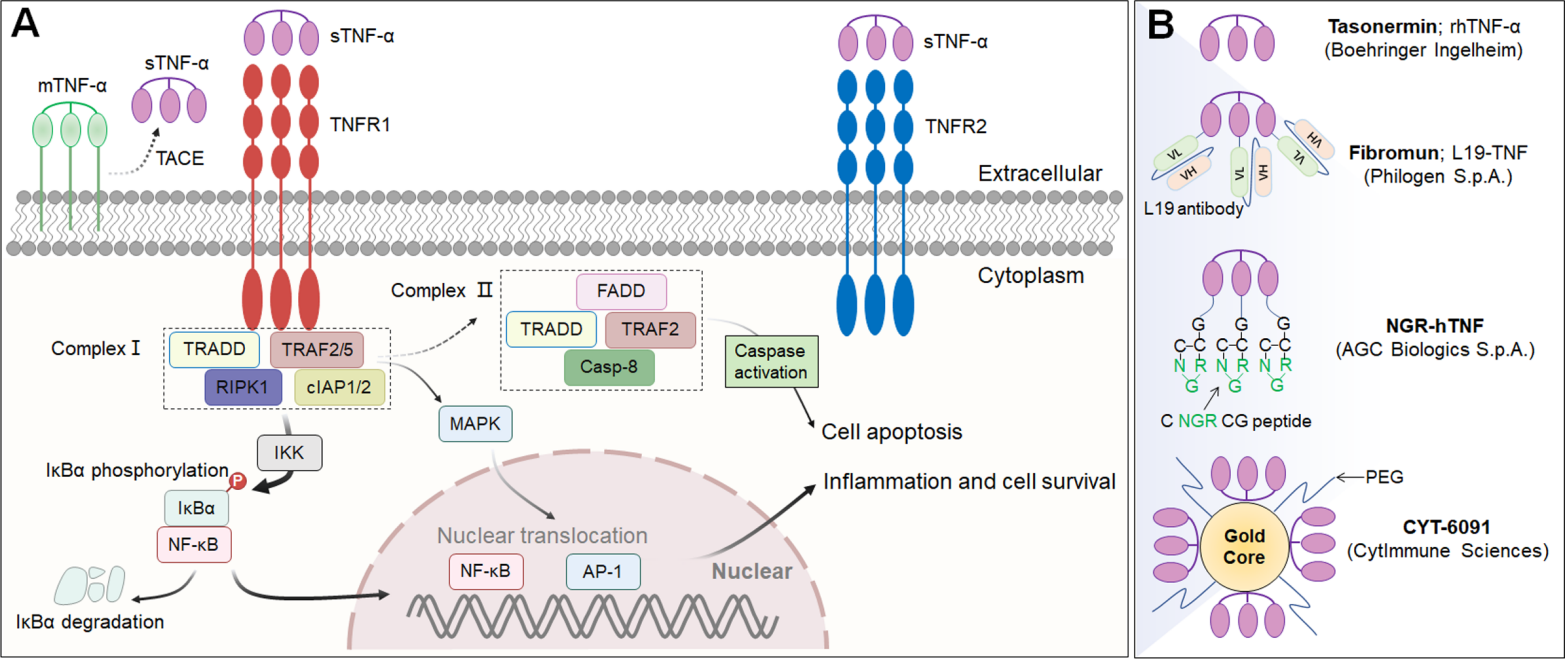
The cascade of the TNF-α signaling pathway and downstream effector molecules and representative therapeutic candidates. **(A)** TNF-α signaling pathway and downstream effector molecules. Cross-linking of soluble TNF-α with TNFR1 primarily triggers proinflammatory pathways that activate NF-κB, MAPK and antiapoptotic response factors, followed by the production of other proinflammatory cytokines. **(B)** Representative therapeutic candidates for engineered TNF-α. TACE: Tumor necrosis factor (TNF)-alpha converting enzyme; mTNF-α: transmembrane TNF-α; sTNF-α: soluble TNF-α.

Tumor necrosis factor alpha (TNF-α; also called TNF or TNFSF2) is a homotrimeric proinflammatory cytokine in the TNF superfamily and a multifunctional molecule involved in regulating important biological processes, including the induction of apoptotic cell death and inflammatory immune responses and the inhibition of tumorigenesis and viral replication (189). It is predominantly synthesized by activated monocytes/macrophages as a 26 kDa transmembrane protein that can be enzymatically cleaved to produce a 17 kDa soluble TNF form (189). Notably, TNF-α is unique in that it activates biological effects at two distinct receptors—TNF receptor type 1 (TNFR1; CD120a; TNFR60) and TNF receptor type 2 (TNFR2; CD120b; TNFR80) (190). Recent evidence suggests that TNFR1 is commonly expressed on almost all cells, whereas TNFR2 expression is limited largely to Treg cells (191). Additionally, cross-linking of soluble TNF-α with TNFR1 primarily triggers proinflammatory pathways that activate NF-κB, MAPK and antiapoptotic response factors and subsequently produce other proinflammatory cytokines, such as IL-1, IL-6 and GM-CSF. Nevertheless, transmembrane TNF binding to TNFR2 typically activates different signaling pathways than those activated by TNFR1, initiating immune modulation and tissue regeneration (192) (**Figure 7A**).

TNF-α is considered to be an effective tumoricidal cytokine because of its capacity to directly induce apoptosis and stimulate the inflammatory response at tumor sites. More importantly, recombinant TNF-α (rTNF-α) can rapidly destroy tumor blood vessels, eliminate hypertension inside tumors, disrupt tumor tissue growth, and activate CD8^+^ T cells. However, systemic administration of a clinically relevant dose of rTNF-α, that is, a dose greater than 300 μg/m^2^ has been limited due to substantial toxic side effects (193). Hence, the early clinical applications of rTNF-α have been restricted to localized regional uses.

**Tasonermin** (Beromun^®^; developed by Boehringer Ingelheim), an agent approved for use by the European Medicine Agency (EMA) in 1999, is a human recombinant form of soluble TNF-α produced in *Escherichia coli* cell culture that can kill cancer cells directly, destroy the blood vessels that supply tumors with nutrients and oxygen, and activate the immune system to fight tumor vascular structure (**Figure 7A**). Tasonermin is available for the treatment of patients with soft tissue sarcoma in combination with melphalan and is administered via isolated limb perfusion (ILP) to minimize systemic exposure (194). The recommended dose is 3 mg in the arm or 4 mg in the leg administered over 90 minutes.

Recombinant human TNF-α (rhTNF-α) and thiolated PEG were concurrently bound to the surface of 27 nanometer colloidal gold particles to generate **CYT-6091** (Aurimune™), a revolutionary tumor-targeting nanomedicine (**Figure 7B**) (195). Preclinical studies have shown that CYT-6091 was enriched in tumors 6 hours after intravenous injection due to enhanced permeability and retention (EPR) effects inside the tumor, and a negligible amount of CYT-6091 was taken up by the liver or spleen (196). In a Phase I clinical study, Cytimmune Sciences disclosed the ability of CYT-6091 to deliver 4-fold the MTD of native TNF-α with no dose limiting toxicities (NCT00356980; NCT00436410). Furthermore, a clinical trial agreement has been signed with the National Cancer Institute (NCI) for establishing a Phase II trial to assess CYT-6091 effectiveness in combination with nab-paclitaxel (Abraxane) in participants with late stage endocrine tumors of the pancreas and thyroid.

Concurrently, a number of TNF-α fusion proteins have been created to guide TNF-α towards tumor tissue by incorporating targeting domains that recognize tumor or tumor-related stromal cell markers. **Fibromun** (L19-TNF) is a fully human immunomodulatory agent consisting of a human anti-L19 antibody in the scFv format linked to the proinflammatory cytokine TNF-α, which yields a stable noncovalent trimetric L19-TNF immunocytokine (**Figure 7B**) (197). Based on encouraging preclinical data, L19-TNF is being studied in numerous Phase I/II clinical trials for the treatment of advanced or metastatic soft tissue sarcoma in combination with doxorubicin (NCT04650984; NCT04733183) and for the treatment of glioblastoma in combination with lomustine (NCT04573192) or temozolomide chemoradiotherapy (NCT04443010) (198). In addition, several Phase II studies on the intratumoral administration of L19-IL2 plus L19-TNF in patients with metastatic melanoma (NCT02076633) or nonmelanoma skin cancer (NCT04362722; NCT05329792) are ongoing (199).

Another TNF-α fusion protein under clinical investigation is **NGR-hTNF**, in which homotrimeric TNF-α is fused to the C-terminus of a cyclic CNGRCG peptide, which targets a CD13 (aminopeptidase N) isoform expressed on angiogenic blood vessels (**Figure 7B**) (200). Recent preclinical results suggested that CNGRC binding to CD13 not only determined the homing specificity of NGR-hTNF to tumor vessels but also reduced the activation of prosurvival pathways and increased the activation of caspases (200). NGR-hTNF is in an ongoing Phase III clinical study for the treatment of advanced malignant pleural mesothelioma (NCT01098266), but recent data have suggested that the study did not meet primary endpoints because patients whose disease progressed rapidly after first-line therapy had a poor prognosis (201). Additionally, NGR-hTNF is also being investigated alone or in combination with chemotherapy in five other randomized Phase II studies for the treatment of participants with ovarian cancer (NCT03804866; NCT01358071), CRC (NCT00483080), hepatocellular carcinoma (NCT00484211), small cell lung cancer (NCT00483509; NCT00994097), and metastatic adult soft tissue sarcoma (NCT00484341).

Although their bioactivity is increased upon targeting, most TNF-α-based immunocytokines induce a notable risk of adverse side effects because of the potential off-target effects of the active TNF-α effector moiety. Accordingly, to minimize the risk of off-target effects and improve the safety of highly bioactive and potentially toxic TNF-α fusion proteins, researchers have sought to develop TNF-α-based prodrugs. For example, Klaus Pfizenmaier and team from the University of Stuttgart developed a TNF-α-based prodrug fusion protein composed of a FAP antibody scFv fragment specific to a target antigen, a TNF-α homotrimeric module, a protease-sensitive linker and a masking fragment from the TNFR1-driven extracellular domain (ECD) (202). To overcome the known heterogeneity of protease expression, the authors constructed two effective protease-sensitive linkers, matrix metalloproteinase-2 (MMP-2) and urokinase-type plasminogen activator (uPA), both of which exhibited effective proteolysis with the respective recombinant protease and led to greatly increased target antigen-independent TNF-α bioactivity (203).

## Conclusions and perspectives

Cancer immunotherapy, represented by immune checkpoint monoclonal antibodies, provides favorable tools for reinvigorating effector immune cells, but the vast majority of cancer patients (more than 80%) do not benefit from these immunotherapies due to limited responsiveness and primary and acquired drug resistance. Strikingly, cytokines can overcome the related resistance mechanisms because of their potential to expand and reactivate effector T and NK cells and to enhance lymphocyte infiltration, in addition to their persistence in the TME (8). However, as a class of complex immune mediators, the use of cytokines as therapeutic agents faces huge challenges, including the difficulty of large-scale manufacturing, which requires increased understanding of cytokine biology and the use of modern biotechnology to develop their antitumor activity while minimizing toxicity. Large industrial companies, risk-taking startups, and academic laboratories have all extensively invested in basic research aimed at engineering and manufacturing cytokine drugs, which has led to a significant resurgence in ongoing clinical trials, and the results are eagerly anticipated.

For future cytokine-based drug development, multiple crucial challenges should be considered: (i) overcoming the naturally quick clearance and poor exposure of endogenous cytokines, (ii) concentrating the delivery of cytokines to diseased tissues or particular cell types to prevent systemic toxicity, and (iii) developing combination regimens to achieve synergistic effects with immune checkpoint blockade antibodies, CAR-T therapy, or gene therapy. To date, many strategies have been employed to meet clinical needs, including half-life extension, antigen targeting, conditional activation, potency manipulation and local delivery. These emerging strategies allow fine-tuning of cytokine signaling pathways and a tailored cellular response. Accordingly, based on current clinical data and progress of immunocytokine-based therapeutics, the IL-15 agonist N-803 (Anktiva™) is currently the only one that submitted a BLA application to the FDA for NMIBC and is more likely to be successful in this indication or beyond in terms of clinical efficacy and might also work in other formats e.g., with antibody conjugation. Therefore, IL-15-related immunocytokine therapeutics would be an important direction for future industrial development suitable for clinical use, although it now faces some challenges related to chemical, manufacturing and control (CMC) development and would more likely to be addressed in the near future. The key to using cytokines as therapeutic agents is to improve therapeutic windows either by reducing potency applying mutational design by protein engineering or by tissue/tumor targeted delivery or the combination of the two strategies. The other direction might be tissue/tumor-specific activation of cytokines by applying prodrug strategies that render tumor-specific cleavage of masking peptide, scFv, or natural binding receptors conjugated to the binding regions of immunocytokines. Thus, potency-reduced cytokines conjugated with antibodies for tumor targeting or cytokine-based prodrug strategies may more likely lead to the development of new immunotherapeutics suitable for future clinical use. Additionally, immunogenicity is an intractable problem for all mutant and conjugated nonendogenous proteins, and methods to reduce immunogenicity also need to be considered in future developments.

Most importantly, a deeper understanding of the biology and intricate cellular processes of cytokines in the TME, determined using approaches such as cryo-electron microscope techniques, single-cell RNA sequencing, transcriptome analysis, and preclinical mechanistic studies, will help shed light on the currently unknown biological activity of cytokines. Concurrently, molecular designs based on contemporary protein engineering technologies to fine-tune cytokine biology, deconvolute the pleiotropism of cytokines, enhance cytokine drug-like properties, and drive selective proinflammatory effects will further facilitate the clinical translation of cytokine-based therapeutics to enhance cancer immunotherapy.

## Author contributions

YF designed and wrote the manuscript. XZ gave the original idea and design. XZ and RT confirmed the final release.

## Glossary

**Table:**

ILs	interleukins
IFNs	interferons
TNF	tumor necrosis factor
TME	tumor microenvironment
RT	radiotherapy
IFNα-2b	interferonα-2b
FDA	U.S. Food and Drug Administration
RCC	renal cell carcinoma
PEG	polyethylene glycol
BLA	license application
NMIBC	nonmuscle-invasive bladder cancer
BCG	bacillus Calmette-Guerin
PK	pharmacokinetics
HSA	human serum albumin
Fc	fragment crystallizable region
IgG	immunoglobulin
PD	pharmacodynamics
VH	variable heavy
VL	variable light
scFv	single-chain variable fragment
FcRn	neonatal Fc receptor for IgG
Fcγ R	Fcγ receptors
ADCC	antibody-dependent cellular cytotoxicity
ADCP	antibody-dependent cellular phagocytosis
CDC	complement-dependent cytotoxicity
NK	natural killer
Treg	regulatory T
ILC2s	group 2 innate lymphoid cells
PFS	progression-free survival
ORR	overall response rate
FIH	first-in-human
TAA	tumor-associated antigen
EpCAM	epithelial cell adhesion molecule
GD2	ganglioside D2
CEA	carcinoembryonic antigen
ECM	extracellular matrix
EDA	extra domain A
EDB	extra domain B
MTD	maximal tolerated dose
FAP	fibroblast activating protein
IL2v	IL-2 variant
DLBCL	diffuse large B-cell lymphoma
dsDNA	double-stranded DNA
ssDNA	single-stranded DNA
HLA	human leukocyte antigen
CR	complete response
DFS	disease-free survival
R/R	relapsed/refractory
NHL	non-Hodgkin's lymphoma
MM	multiple myeloma
CRC	colorectal cancer
SCCHN	squamous cell carcinoma of the head and neck
IL-15Ra	IL-15 receptor alpha
CTCL	cutaneous T-cell lymphoma
hetIL-15	heterodimeric IL-15
DLTs	dose-limiting toxicities
IND	Investigational New Drug Application
AML	acute myelogenous leukemia
MDS	myelodysplastic syndrome
MDSCs	myeloid derived suppressor cells
T<sub>SCM</sub>	stem cell-like memory T cells
CRS	cytokine release syndrome
DCs	dendritic cells
Tfh	T follicular helper
Th17	T helper 17
PR	partial response
TIGIT	T-cell immunoreceptor with Ig and ITIM domains
TAMs	tumor-associated macrophages
CEA. Tg	carcinoembryonic antigen transgenic
TKI	tyrosine kinase inhibitor
CAR-T	Chimeric antigen receptor T
IL-15Rα Su	soluble domain of IL-15Rα
NMPA	National Medical Products Administration
ISGs	interferon-stimulated genes
ILP	isolated limb perfusion
EPR	enhanced permeability and retention
NCI	National Cancer Institute
ECD	extracellular domain
uPA	urokinase-type plasminogen activator
MMP-2	matrix metalloproteinase-2
